# Structural model of human dUTPase in complex with a novel proteinaceous inhibitor

**DOI:** 10.1038/s41598-018-22145-8

**Published:** 2018-03-12

**Authors:** Kinga Nyíri, Haydyn D. T. Mertens, Borbála Tihanyi, Gergely N. Nagy, Bianka Kőhegyi, Judit Matejka, Matthew J. Harris, Judit E. Szabó, Veronika Papp-Kádár, Veronika Németh-Pongrácz, Olivér Ozohanics, Károly Vékey, Dmitri I. Svergun, Antoni J. Borysik, Beáta G. Vértessy

**Affiliations:** 1 Department of Applied Biotechnology and Food Sciences, Budapest University of Technology and Economics, Budapest, 1111 Hungary; 2 Institute of Enzymology, Research Centre for Natural Sciences, Hungarian Academy of Sciences, Budapest, 1117 Hungary; 3 European Molecular Biology Laboratory, Hamburg Outstation, c/o DESY, Notkestrasse 85, Hamburg, 22603 Germany; 4 Department of Chemistry, King’s College London, Britannia House, London, SE1 1DB United Kingdom; 5 Institute of Organic Chemistry, Research Centre for Natural Sciences, Hungarian Academy of Sciences, Budapest, 1117 Hungary; 6 Present Address: Division of Structural Biology, University of Oxford, Roosevelt Drive, Oxford, OX3 7BN United Kingdom

## Abstract

Human deoxyuridine 5′-triphosphate nucleotidohydrolase (dUTPase), essential for DNA integrity, acts as a survival factor for tumor cells and is a target for cancer chemotherapy. Here we report that the Staphylococcal repressor protein Stl_SaPIBov1_ (Stl) forms strong complex with human dUTPase. Functional analysis reveals that this interaction results in significant reduction of both dUTPase enzymatic activity and DNA binding capability of Stl. We conducted structural studies to understand the mechanism of this mutual inhibition. Small-angle X-ray scattering (SAXS) complemented with hydrogen-deuterium exchange mass spectrometry (HDX-MS) data allowed us to obtain 3D structural models comprising a trimeric dUTPase complexed with separate Stl monomers. These models thus reveal that upon dUTPase-Stl complex formation the functional homodimer of Stl repressor dissociates, which abolishes the DNA binding ability of the protein. Active site forming dUTPase segments were directly identified to be involved in the dUTPase-Stl interaction by HDX-MS, explaining the loss of dUTPase activity upon complexation. Our results provide key novel structural insights that pave the way for further applications of the first potent proteinaceous inhibitor of human dUTPase.

## Introduction

Fine-tuned regulation of nucleotide metabolism to ensure DNA replication with high fidelity is essential for proper development in all free-living organisms^1–8^. Imbalanced nucleotide pools leading to genomic instability have been implicated in numerous human diseases, e.g. neoplastic and neuronal pathogenesis^9–11^. Hence, dNTP pool sanitizing enzymes have gained much recent interest in biomedical research^4,5,12–16^.

Deoxyuridine triphosphate nucleotidohydrolase (dUTPase, E.C. 3.6.1.23) catalyzes the hydrolysis of dUTP to dUMP and pyrophosphate providing substrate for thymidylate synthase and eliminating dUTP from the DNA biosynthetic pathway^1,17–19^. Deficiency or impaired enzymatic activity of dUTPase leads to the elevation of the intracellular dUTP pool and can lead to enhanced uracil incorporation into the DNA^20,21^. If high genomic uracil content overloads the base-excision repair capacity it will result in DNA fragmentation and subsequent cell death^19,20,22–25^. Proper dUTPase function is thus of key importance in preventing DNA uracilation and hence, in preserving genome integrity.

Uracil misincorporation is also a mechanism of cytotoxicity induced by chemotherapeutic agents which target thymidylate synthase in the treatment of different tumors^15,26^. This approach has been extensively applied in the past few decades, however, with a notable limitation of prevalent drug resistance resulting in a high treatment failure rate^27^. dUTPase overexpression has been shown to be an important mediator of sensitivity to thymidylate synthase targeting agents in various cancer cell lines and tumor specimens and to modulate treatment outcome^9,10,27–31^. These facts underline the significance of dUTPase inhibitory studies to better understand the role dUTPase plays in the process of cell death and characterize this enzyme as a promising additional target for anti-cancer drug development in combination therapies with thymidylate synthase inhibitors^32^. Accordingly, numerous studies provide evidence that siRNA-based silencing^5^ or small molecule (TAS-114) inhibition^33^ of the human dUTPase induces synthetic lethality with fluoropyrimidine (FUdR, 5-FU) and antifolate (pemetrexed) treatments in HeLa, CRC, NSCLC cell line models and enhances the anti-tumor effect of therapeutic drugs in mouse breast cancer (MX-1) xenografts^15^.

In addition to small molecular inhibitors, proteinaceous inhibitors present a promising alternative for blocking enzyme function with increased selectivity and, consequently, with less intracellular off-target effects. Such protein inhibitors also allow specific disruption of cellular DNA repair pathways, facilitating *in cellulo* studies on the effect of enzyme inhibition. A prominent example is presented by the uracil-DNA glycosylase inhibitor (UGI) that binds to and blocks the activity of the major uracil-DNA glycosylase enzyme (UNG)^34,35^. UGI has been first isolated from the bacteriophage PBS2 and it has also been successfully used to provide potent inhibitor against UNGs from various sources, including the human enzyme^34–38^.

In a similar approach, recent identification of a novel kind of bacteriophage dUTPase inhibitor protein envisioned additional key opportunities for pathway-specific perturbation of thymidylate biosynthesis. Namely, Stl_SaPIBov1_ (Stl), a pathogenicity island (SaPI) repressor protein in *Staphylococcus aureus*, has been shown to interact with the *Φ11*, 80*α* and φNM1 helper phage dUTPases^29,39^. This interaction results in the disruption of Stl-DNA complex which allows the transcription of the repressed PI genes and the excision and replication of the SaPI^40,41^. Remarkably, the catalytic activity of *Φ11* dUTPase (Φ11DUT) is also inhibited upon complex formation with Stl, due to the competition of Stl and the substrate dUTP for dUTPase binding revealed by transient kinetic analysis^42^. Moreover, in our recent studies we have also demonstrated that Stl acts as a potent inhibitor of the *Mycobacterial* dUTPase, both *in vitro* and *in vivo*^21^ and exerts inhibitory effect on the *Drosophila melanoglaster* dUTPase *in vitro*^43^.

In the present study, we investigated the potential of Stl to act as an inhibitor of the human dUTPase (hDUT) using various biochemical and biophysical methods. We found that Stl forms a tight complex with hDUT *in vitro* which results in a strong inhibition of hDUT enzymatic activity. Based on these results we propose that Stl may be considered as a potent cross-species protein inhibitor of the human dUTPase enzyme. Understanding the molecular mechanism of Stl action on dUTPase might constitute the first step towards the design of an efficient and selective proteinaceous inhibitor.

Towards this end, here we present detailed structural insights on this protein-protein interaction with an integrated structural biology approach, based on a cutting-edge combination of ITC, native mass spectrometry, and most prominently size-exclusion chromatography in line with small-angle X-ray scattering (SEC-SAXS) and hydrogen-deuterium exchange mass spectrometry (HDX-MS). While SAXS has already been at the forefront of structural biology when applied to complexes that fail to crystallize, structure-related information from HDX-MS has only recently been exploited^44,45^. Recent examples show that combination of the increasingly powerful HDX-MS and SEC-SAXS techniques substantiate advanced 3D models^46^. The structural insights provided by the present work may serve as the first step towards the design of an efficient and selective proteinaceous inhibitor of the human dUTPase which can be applied as a cellular tool for *in vivo* focusing on the physiological function of dUTPase.

## Materials and Methods

Materials for electrophoresis or chromatography were from BioRad or Amersham Biosciences, molecular biology products were from New England Biolabs or Fermentas. Other materials were from Merck KGaA, if not indicated otherwise.

### Protein expression and purification

Proteins in this study were expressed in *E*. *coli* BL21 (DE3) Rosetta cells with 0.5 l LB medium and being induced by 0.5 mM isopropyl β-D-1-thiogalactopyranoside IPTG at OD_600_ = 0.6. Stl (orf 20 of SaPI_bov1_, GI: 11094374) was expressed from a pGEX-4T-1 vector^42^ (see Supporting information), for 4 h at 30 °C. The nuclear isoform of human dUTPase was expressed from a pET15b vector^47^ for 4 h at 37 °C. Finally, cells were harvested by centrifugation and stored at −80 °C.

Purification of Stl and hDUT was performed as described previously^22,42^ (see Supporting information). All protein preparations were at least 95% pure as judged by SDS–PAGE. Concentration of proteins were measured by a NanoDrop 2000 UV-Vis spectrophotometer using the 280 nm extinction coefficient 35760 M^−1^ cm^−1^ and 10430 M^−1^ cm^−1^ for Stl and hDUT respectively, calculated based on amino acid composition (http://web.expasy.org/protparam). All protein concentrations throughout this study correspond to protomers.

### Native gel electrophoresis

Native polyacrylamide gel electrophoresis was performed using 8% polyacrylamide gels as described previously^42^. Briefly, after 1 h of pre-electrophoresis at 100 V, 25 μl of pre-mixed samples were loaded on the gel and electrophoresis was done for 5 h at 150 V in Tris-HCl buffer (pH 8.5). The apparatus was chilled on ice to avoid heat-induced denaturation. The gel was stained with Coomassie-Brilliant Blue dye.

### Isothermal titration calorimetry (ITC)

ITC experiments were carried out at 20 °C on a Microcal ITC_200_ instrument (Malvern Instruments, Malvern, UK). Prior to the measurements hDUT and Stl samples were dialyzed overnight at 4 °C in a buffer of 20 mM HEPES (pH 7.5), 300 mM NaCl, 5 mM MgCl_2_, 1 mM TCEP. In the experimental setup, the cell of the instrument was filled with Stl and the syringe with hDUT. The titrations were performed with the injection syringe rotating at 750 r.p.m. and included a series of 20 injections spaced 180 s apart from each other with injection volumes of 0.5 μl for the first titration and 2 μl for the subsequent 19 titrations. The apparent dilution heat observed at the end of the titration was subtracted from the integrated heat data. The corrected data were then analyzed using MicroCal ORIGIN 7.5 software, following the directions of the manufacturer, in order to determine the dissociation constant (K_d_), stoichiometry (N), enthalpy (ΔH) and entropy (ΔS). The best fit to the data was obtained using the one set of independent sites binding model of the software. Average and standard deviation (SD) of the fitted parameters were calculated from three parallel measurements.

### Electrospray ionization mass spectrometry

The protein complexes were studied using an unmodified commercial Waters QTOF Premier mass spectrometer (Waters, Milford, MA, USA) equipped with electrospray ionization source (Waters, Milford, MA, USA) operated in positive ion mode. Mass spectra were obtained under native conditions: namely, the ions were generated from aqueous 5 mM NH_4_HCO_3_ buffer solution (pH 7.5) containing the human dUTPase, Stl or both protein constructs at ca. 0.5 µM monomer concentration. These conditions are favorable for the transfer of the protein complexes from solution into the gas phase. The capillary voltage was 2800 V, the sampling cone voltage was 125 V and the temperature of the source was kept at 80 °C, collision cell pressure was 3.43*10^−3^ mbar and ion guide gas flow was 35.00 ml/min. Mass spectra were recorded using the software MassLynx 4.1 (Waters, Milford, MA, USA) in the 1000–8000 m/z mass range.

### dUTPase activity assay

Proton release during the hydrolysis of dUTP into dUMP and PP_i_ was followed continuously at 559 nm at 20 °C using a Specord 200 spectrophotometer (Analytic Jena, Germany) and 10 mm path length thermostatted cuvettes as described previously^21,42,48^. Reaction mixtures contained 50 nM hDUT enzyme and varying concentrations of Stl in activity buffer (1 mM HEPES (pH 7.5), 5 mM MgCl_2_, 150 mM KCl, 40 μM Phenol Red indicator). The reaction started with the addition of 30 μM dUTP after 5 min pre-incubation of the two proteins in the assay buffer. Initial velocity was determined from the slope of the first 10% of the progress curve. Stl inhibition data were fitted to the quadratic binding equation describing 1:1 stoichiometry for the dissociation equilibrium with no cooperativity^42^.

### Electrophoretic mobility shift assay (EMSA)

EMSA experiments were performed to test the effect of hDUT addition on the interaction of Stl_SaPIBov1_ to its predicted DNA binding site within *S*. *aureus*^49^. For this a 43 bp double stranded oligonucleotide (previously named as interR) was used^49^. This oligonucleotide contains the symmetric Stl DNA binding site located in the intergenic region between the *stl* and the *str* genes. The two strands of this oligonucleotide (5′-ATGTTGAAATAAATATCTCGATGTGAGATAATTTGTTCGAGGA-3′ and 5′-TCCTCGAACAAATTATCTCACATCGAGATATTTATTTCAACAT-3′) were custom synthesized by Eurofins MWG Operon and hybridized by controlled gradual cooling after 5 min incubation on 95 °C. Proteins were mixed with 100 ng DNA in 20 μl total volume (NaCl concentration 100 mM in all samples). After 15 min incubation at 4 °C, samples were loaded onto a polyacrylamide gel (8%). Electrophoresis was performed in Tris-Borate-EDTA (TBE) buffer for 70 min at room temperature (150 V), following 1 h pre-electrophoresis of the gel (100 V). Bands were detected with an Uvi-Tec gel documentation system (Cleaver Scientific Ltd., Ruby, UK) after staining with GelRed (Bioticum).

### Hydrogen deuterium exchange mass spectrometry (HDX-MS)

HDX-MS experiments were performed on a Synapt G2Si HDMS coupled to an Acquity UPLC M-Class system with HDX and automation (Waters Corporation, Manchester, UK). The isotope uptake of the human dUTPase and Stl proteins was determined using a continuous labelling workflow at 20 °C. Briefly, each protein was dissolved in buffer E (20 mM HEPES, 300 mM NaCl, 5 mM MgCl_2_, pH 7.5) to a final concentration of 10–20 µM. Isotope labelling was initiated by diluting 5 μl of each protein into 95 μl of buffer L (20 mM HEPES, 300 mM NaCl, 5 mM MgCl_2_ in D_2_O, pD 7.1). Samples were quenched in ice cold buffer Q (2.4% formic acid) after various incubation times then being digested online with a Waters Enzymate BEH pepsin column at 20 °C. The peptides were trapped on a Waters BEH C18 VanGuard pre-column for 3 minutes at a flow rate of 200 μl/min in buffer A (0.1% formic acid ~pH 2.5) and then applied to a Waters BEH C-18 analytical column. Peptides were eluted with a linear gradient of buffer B (0.1% formic acid in acetonitrile ~pH 2.5) at a flowrate of 40 μl/min. All trapping and chromatography was performed at 0.5 °C to minimize back exchange. Characterization of the bound HDX behavior of each protein was carried out by premixing the proteins at equimolar concentrations. MS data were acquired using an MS^E^ workflow in HD mode with extended range enabled to reduce detector saturation and maintain peak shapes. 6 reference acquisitions were obtained for each protein along with labelling acquisitions of 1, 10 and 100 minutes which were obtained in triplicate. The MS was calibrated separately against NaI and the MS data were acquired with lock mass correction using Leu-enkephalin.

Peptides were assigned with the ProteinLynx Global Server (PLGS, Waters Corporation, Manchester, UK) software and the isotope uptake of each peptide was determined with DynamX v3.0. Evaluation of data fitting and error of each dataset were performed as described previously^50^. The total mass shift of each peptide was then plotted against the residue position to generate Woods plots showing the Δmass of each peptide in the bound state^51^. The average mass shift across all peptides at each residue was then calculated and any values that exceed the 95% confidence bands for any residue were noted and defined as interaction surfaces of the two proteins. Difference plots (Δmass) were prepared for each protein by subtraction of the bound from the unbound HDX-MS patterns of each protein. We assumed that all subunits were involved in the protein-protein interactions such that the interacting surfaces were projected equally across all subunits.

### Small angle X-ray scattering

#### SAXS measurements

Synchrotron radiation X-ray scattering data were collected on the EMBL P12 beamline of the storage ring PETRA III (DESY, Hamburg) (Tables 1 and 2), using a PILATUS 2 M pixel detector (DECTRIS, Switzerland). For batch experiments of hDUT and Stl, samples were measured in buffer B (50 mM HEPES, pH = 7.5, 300 mM NaCl, 5 mM MgCl_2_) while flowing through a temperature controlled capillary (1.2 mm I.D.) at 20 °C and 20 frames of 0.05 s exposure time were collected. hDUT:Stl complexes (100 µL at ~8 mg/ml) were injected onto a GE Healthcare S200 Increase 10/300 (24 ml) column equilibrated in buffer B and in-line SEC-SAXS performed at a flow rate of 0.5 ml/min. A total of 3600 × 1 second SAXS data frames were recorded during elution.

**Table 1:** SAXS Data collection and derived parameters for hDUT and Stl. Abbreviations: *M*_*r*_: molecular mass; *R*_*g*_: radius of gyration; *D*_*max*_: maximal particle dimension; *V*_*p*_: Porod volume; *V*_*ex*_: Particle excluded volume.

	hDUT	Stl
**Data collection parameters**		
Instrument	EMBL P12 beam line (PETRA-III, DESY, Hamburg)	
Beam geometry	0.2 × 0.12 mm^2^	
Wavelength (Å)	1.24	
*s* range (Å^−1^)^a^	0.01–0.46	
Exposure time (s)	1 (20 × 0.05 s)	
Concentration range (mg/mL)	0.2–2.0	0.2–0.9
Temperature (K)	283	283
**Structural parameters** ^**b**^		
*I*(*0*) (cm^−1^) [from *p*(*r*)]	0.025 ± 0.001	0.032 ± 0.001
*R*_*g*_ (Å) [from *p*(*r*)]	29 ± 1	32 ± 1
*I*(*0*) (cm^−1^) (from Guinier)	0.025 ± 0.001	0.031 ± 0.001
*R*_*g*_ (Å) (from Guinier)	30 ± 1	33 ± 1
*D*_*max*_ (Å)	100	105
Porod volume estimate (Å^3^)	117000 ± 10000	100000 ± 20000
Excluded volume estimate (Å^3^)	132000 ± 20000	90000 ± 20000
Dry volume calculated from sequence (Å^3^)^c^	21815	38738
**Molecular-mass determination**		
*I*(*0*) (cm^−1^) BSA (70,000 Da)	0.050 ± 0.001	
Molecular mass *M*_r_ (Da) [from *I*(*0*)]	35000 ± 4000	45000 ± 5000
Molecular mass *M*_r_ (Da) [from Porod volume (*V*_*p*_*/1*.*6*)]	73000 ± 8000	59000 ± 6000
Molecular mass *M*_r_ (Da) [from excluded volume (*V*_*ex*_*/2*)]	66500 ± 10000	45000 ± 5000
Calculated monomeric *M*_r_ from sequence (Da)	18029	32015
**Software employed**		
Primary data reduction	RADAVER	
Data processing	PRIMUS/Qt	
Ab initio analysis	DAMMIF	
Validation and averaging	DAMAVER	
Hybrid modeling	CORAL, EOM	
Computation of model intensities	CRYSOL	
3D graphics representations	PyMOL, UCSF Chimera	

^a^Momentum transfer *s* = 4πsin(θ)/λ. ^b^Values reported for merged data sets (0.2 & 2.0, and 0.2 & 0.9 mg.mL^−1^ for hDUT and Stl, respectively). ^c^Dry volume determined using the server: http://www.basic.northwestern.edu/biotools/proteincalc.html.

**Table 2:** SAXS Data collection and derived parameters for hDUT:Stl SEC-SAXS. Abbreviations: *M*_*r*_: molecular mass; *R*_*g*_: radius of gyration; *D*_*max*_: maximal particle dimension; *V*_*p*_: Porod volume; *V*_*ex*_: Particle excluded volume.

	hDUT:Stl (Region 1)	hDUT:Stl (Region 2)
**Data collection parameters**		
Instrument	EMBL P12 beam line (PETRA-III, DESY, Hamburg)	
Beam geometry	0.2 × 0.12 mm^2^	
Wavelength (Å)	1.24	
*s* range (Å^−1^)^a^	0.01–0.46	
Exposure time (s)	3600 (3600 × 1.0 s)	
Concentration range (mg/mL)	~0.4	~0.4
Temperature (K)	283	283
**Structural parameters** ^**b**^		
*I*(*0*) (cm^−1^) [from *p*(*r*)]	0.041 ± 0.001	0.047 ± 0.001
*R*_*g*_ (Å) [from *p*(*r*)]	45 ± 1	39 ± 1
*I*(*0*) (cm^−1^) (from Guinier)	0.041 ± 0.001	0.046 ± 0.001
*R*_*g*_ (Å) (from Guinier)	44 ± 1	38 ± 1
*D*_*max*_ (Å)	170	140
Porod volume estimate (Å^3^)	227000 ± 10000	190000 ± 20000
Excluded volume estimate (Å^3^)	290000 ± 2000	242000 ± 20000
Dry volume calculated from sequence (Å^3^)^c^	188384/149667 (3:3/3:2 complex)	
**Molecular-mass determination**		
*I*(*0*) (cm^−1^) BSA (70,000 Da)	0.050 ± 0.001	
Molecular mass *M*_r_ (Da) [from *DATMOW*]	130000 ± 4000	124000 ± 5000
Molecular mass *M*_r_ (Da) [from Porod volume (*V*_*p*_*/1*.*6*)]	134000 ± 9000	112000 ± 6000
Molecular mass *M*_r_ (Da) [from excluded volume (*V*_*ex*_*/2*)]	145000 ± 10000	121000 ± 3000
Calculated monomeric *M*_r_ from sequence (Da)	155689/123692 (3:3/3:2 complex)	
**Software employed**		
Primary data reduction	RADAVER	
Data processing	PRIMUS/Qt	
Ab initio analysis	DAMMIF	
Validation and averaging	DAMAVER	
Hybrid modeling	CORAL	
Computation of model intensities	CRYSOL	
3D graphics representations	PyMOL, UCSF Chimera	

^a^Momentum transfer *s* = 4πsin(θ)/λ. ^b^Values reported for selected buffer corrected SEC-SAXS frames. ^c^Dry volume determined using the server: http://www.basic.northwestern.edu/biotools/proteincalc.html.

The sample-to-detector distance was 3.1 m, covering a range of momentum transfer 0.01 Å^−1^ ≤ s ≥ 0.46 Å^−1^ (s = 4πsinθ/ λ, where 2θ is the scattering angle, and λ = 1.24 Å is the X-ray wavelength). Based on comparison of successive frames, no detectable radiation damage was observed. Data from the detector were normalized to the transmitted beam intensity, averaged, placed on absolute scale relative to water and the scattering of buffer solutions subtracted. All data manipulations were performed using PRIMUS*qt* and the ATSAS software package^52^. SEC-SAXS data were analyzed using CHROMIXS^53^.

The forward scattering *I*(*0*) and radius of gyration, *R*_*g*_ were determined from Guinier analysis^54^ assuming that at very small angles (s ≤ 1.3/*R*_*g*_) the intensity is represented as *I*(*s*) = *I*(*0*)exp(−(*sR*_*g*_)2/3)). These parameters were also estimated from the full scattering curves using the indirect Fourier transform method implemented in the program GNOM^55^, along with the distance distribution function *p*(*r*) and the maximum particle dimensions *D*_*max*_. Molecular masses (*MMs*) of solutes were estimated from SAXS data by comparing the extrapolated forward scattering with that of a reference solution of bovine serum albumin (Merck KGaA, Darmstadt, Germany), the hydrated-particle/Porod volume *V*_*p*_, where molecular mass is estimated as 0.588 times *V*_*p*_, and from the excluded solvent volumes, *V*_*ex*_ obtained from *ab initio* modeling in the program DAMMIF^56^. *MMs* from in-line SEC-SAXS data were also estimated using the DATMOW^57^ routine implemented in ATSAS. Computation of theoretical scattering intensities was performed using the program CRYSOL^58^.

#### Ab initio shape determination

Low resolution shapes were reconstructed from SAXS data using the programs DAMMIF^56^, which represents the macromolecule as a densely packed interconnected configuration of beads or chain-like ensemble of dummy residues, respectively, that best fits the experimental data *I*_*exp*_(*s*) by minimizing the discrepancy according to equation (1), where *N* is the number of experimental points, *c* is a scaling factor and *I*_*calc*_(*s*_*j*_) and *σ*(*s*_*j*_) are the calculated intensity and the experimental error at the momentum transfer *s*_*j*_, respectively.

1 $${\chi }^{2}=\frac{1}{N-1}\sum _{j}{[\frac{{I}_{\exp }({s}_{j})-c{I}_{calc}({s}_{j})}{\sigma ({s}_{j})}]}^{2}$$

For both 3:3 and 3:2 hDUT:Stl complex data models were generated initially without symmetry (P1) and then enforcing P3 (3:3) or P2 (3:2). The symmetry constrained models were checked to be consistent with the P1 reconstructions through superposition in SUPALM^59^. As a quantification of the reliability of models, we considered effective resolution determined by the recently published Fourier shell correlation (FSC) approach^60^ and normalized spatial discrepancy^61^ (NSD) values, which represent the measure of the real-space variation of the models. Ideal superposition of two compact and rigid structures with the same low resolution shapes gives NSD around 1. For the models containing flexible regions of significant length, e.g. flexible N/C termini like in the reported case, can still be considered similar in the overall shape if NSD <3.

#### Molecular Modeling

Human dUTPase crystal structures (PDB IDs are 1Q5U^62^ and 3EHW) were used as templates for modeling of the trimeric core of hDUT:Stl complexes. Rigid bodies of Stl were generated applying the protein structure prediction server Phyre2^63^ and dimer models from that were created by M-ZDOCK server^64^ using C-terminal region (T100-N280) of the Stl model as the dimer interface based on our chemical crosslinking results (Supplementary Fig. 3). An additional assessment of Stl protein flexibility based on a dimeric assembly determined using EOM^59^, where a genetic algorithm is used to select an ensemble of best fitting configurations from a randomly generated pool. The program CORAL^52^ was used to perform a multi-step rigid body refinement of hDUT and hDUT:Stl complexes in both 3:3 and 3:2 stoichiometries, where a simulated annealing (SA) based search of subunit arrangements and orientations, stoichiometry and conformations of missing terminal loops was conducted to fit the experimental SAXS data. In the first step, using the SEC-SAXS region 1 data, the position of the hDUT trimer was fixed and ambiguous distance restraints (10 Å) based on the potential contacts identified by hydrogen deuterium exchange mass spectrometry (HDX-MS) defined between each hDUT (residues A37–R44 and L88–H92) and Stl monomer (residues Y98-Y113). For hDUT the interfacial residues identified by HDX-MS were filtered by surface accessibility (cut-off > 10 Å^2^) based on the crystal structure (1Q5U) from the regions with negative HDX signals (H34–L50 and L88–G110). Refinement was conducted in CORAL applying P3 symmetry to generate a symmetric complex with 3:3 hDUT:Stl stoichiometry and to add missing terminal residues and to allow a flexible linker between the N and C-terminal domains of Stl. To generate a 3:2 hDUT:Stl complex, subsequent refinement was performed in CORAL against the major SEC-SAXS peak data (region 2) using the rigid body model of the 3:3 complex as input with one Stl monomer removed and the hDUT:Stl interface fixed.

For modeling based on SAXS data, multiple runs were performed to verify the stability of the solution, and to establish the most typical 3D reconstructions using DAMAVER^65^. SAXS data has been deposited at the SASBDB (www.sasbdb.org) with accession codes: SASDC57, SASDC67, SASDC77, SASDC87.

## Results

### hDUT-Stl complex formation was verified by independent biophysical methods

**Figure 1:**
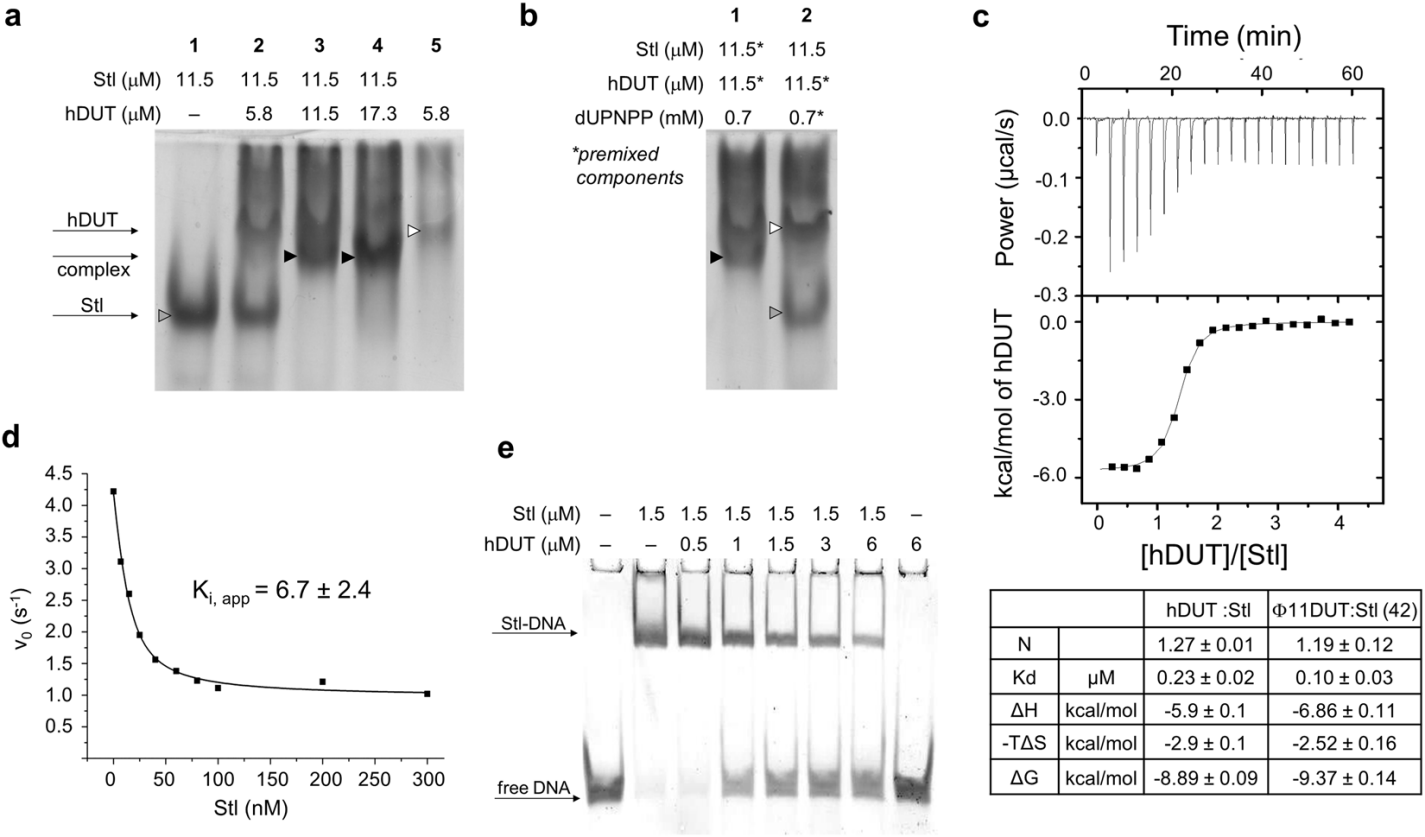
Identification and functional analysis of the interaction between the human dUTPase and Stl. (**A**,**B**) Native gel electrophoresis demonstrates apparence of a band corresponding to hDUT-Stl complex (black arrowhead) when mixing Stl (grey arrowhead) and hDUT (white arrowhead) samples. Panel B shows how the substrate analogue dUPNPP affects complex formation and the dependence of this effect on the order of mixing. (Full-length gel is included in the Supplementary Information.) (**C**) Equilibrium binding of hDUT to Stl assessed by isothermal titration calorimetry (ITC). One representative graph is shown from three parallel experiments. Average and SD of parameters determined by fitting a one set of sites model to the integrated titration data (N, stoichiometry, K_d_, binding affinity, ΔH, binding enthalpy, -TΔS binding entropy, ΔG binding free enthalpy) are shown. Parameters determined from one experiment for Φ11DUT:Stl interaction are shown alongside, errors in that case represent the reliability of the fitting of one set of sites model on the data^42^. (**D**) Inhibitory effects of Stl on hDUT catalytic activity (representative measurement). dUTPase enzymatic activity was measured in the presence and absence of Stl. The quadratic equation line fitted to the data resulted in the apparent K_i_ = 6.7 ± 2.4 nM. (**E**) Stl DNA-binding ability is disrupted during complex formation with hDUT, verified by electrophoretic mobility shift assay (EMSA).(Full-length gel is included in the Supplementary Information.).

To test whether Stl may bind to human dUTPase, we first carried out native polyacrylamide gel electrophoresis (Fig. 1a). We run samples of individual proteins (Fig. 1a, Lanes 1 and 5) and mixtures of the two proteins of 0.5:1, 1:1, 1.5:1 hDUT:Stl molar ratios (Fig. 1a, Lanes 2–4, respectively). Upon mixing of Stl (grey arrowhead) with increasing concentration of hDUT (white arrowhead) in the samples, a distinct additional band (black arrowhead) appeared in parallel with the gradual disappearance of the Stl band, arguing for the formation a stable hDUT:Stl complex (Fig. 1a).

We observed no complex formation when human dUTPase was pre-mixed with 60-fold excess of the slowly hydrolyzable substrate analogue dUPNPP (Fig. 1b, Lane 2). Nevertheless, the pre-formed hDUT:Stl complex did not dissociate upon the addition of the same amount of this substrate analogue (Fig. 1b, Lane 1). These results indicate that Stl is a slow-binding competitor of the substrate analogue, as in the case of Φ11DUT^42^.

We performed additional experiments to quantitatively analyze this Stl-hDUT interaction. Data from isothermal titration calorimetry (ITC) measurements indicated that Stl and hDUT form a considerably strong complex with a K_d_ of 0.23 ± 0.02 µM (Fig. 1c). Note that this apparent affinity is in the same order of magnitude that was determined for the Φ11DUT-Stl interaction with ITC^42^ (cf. Fig. 1c). In addition, thermodynamic analysis displayed a similar energetic contribution in case of hDUT-Stl and Φ11DUT-Stl interactions with favorable contribution of binding enthalpy, and, (to a lesser extent) entropy of complexation. The N = 1.27 ± 0.01 stoichiometry formally indicates that 1.27 hDUT protomer forms a complex with 1 Stl protomer (ie. 3 hDUT protomers, equivalent to a dUTPase trimer interact with to 2.4 Stl protomers). Taking into account that human dUTPase protomers form a stable trimer^66–68^ and considering that Stl is in monomer-dimer equilibrium^42^, this 3 to 2.4 hDUT-Stl protomer ratio could reflect macromolecular complex formation between i) hDUT_3_Stl_2_ formed by one dUTPase trimer and 2 Stl molecules (1 dimer/ 2 monomers) or ii) hDUT_3_Stl_3_ consisting of one hDUT trimer and 3 Stl molecules (1 dimer + 1 monomer/ 3 monomers) or the mixture of these complexes.

To provide additional experimental evidence for the molecular composition of hDUT:Stl complex, we performed native electrospray ionization mass spectrometry (ESI-MS) analysis. In the native MS spectrum of the mixture of the two proteins it was observed that hDUT and Stl form a hetero-oligomer, while peaks of Stl dimer are also present in the spectrum (Supplementary Fig. 1). The molecular mass of the complex was found to be 120.0 ± 0.1 kDa, which corresponds to hDUT_3_Stl_2_ a complex of two Stl monomers (65.8 kDa) with a human dUTPase trimer (54.1 kDa). The peaks of the hetero-oligomer showed a Gaussian-like distribution, with the maximum at m/z 4622 (26+).

### Inhibition of hDUT catalytic activity

Having established that hDUT and Stl form a strong complex, it was of immediate interest to analyze whether this complexation has any effect on the cognate physiological functions of either components of the complex. To test whether complexation with Stl might also affect the function of the human enzyme, we measured hDUT enzymatic activity in the presence and absence of Stl (Fig. 1d). We found that Stl exerted ca. 70% inhibition on human dUTPase catalytic activity (Fig. 1d). Titration experiments with varied concentrations of Stl showed that the inhibitory effect of Stl is associated with an apparent inhibitory constant (K_i_ = 6.7 ± 2.4 nM), suggesting that Stl is a highly potent inhibitor of the human dUTPase. Stl inhibition was only observed when hDUT was pre-incubated with Stl before the addition of dUTP. This indicates that the complex formation between hDUT and Stl is slow as compared to substrate binding, reinforcing the predictions of native polyacrylamide gel electrophoresis results with substrate analogue.

### Perturbation of Stl-DNA complex formation by the human dUTPase

In *Staphylococcus aureus*, Stl acts as a transcriptional repressor for the pathogenicity island SaPI_bov1_ and is responsible for blocking the horizontal transfer of this mobile genetic element^40,41^. We were interested in whether the interaction with hDUT may also be able to modulate Stl repressor activity. To assess this question, electrophoretic mobility shift assay (EMSA) experiments were performed. DNA containing the validated Stl binding site^49^ was added to samples containing Stl, Stl:hDUT complex or hDUT (Fig. 1e). When only Stl was mixed with DNA, the band of DNA was shifted to an upper position of the native polyacrylamide gel because of complex formation (cf. Fig. 1e, Lane 1 and Lane 2). As the hDUT concentration was increased in the samples, intensity of the bands of Stl-DNA complex gradually decreased, meanwhile the bands of free DNA became more intense. These results present clear evidence that complex formation with hDUT inhibited Stl binding to its consensus DNA binding site.

### Analysis of the interaction surface of the human dUTPase: Stl complex by hydrogen deuterium exchange mass spectrometry

**Figure 2:**
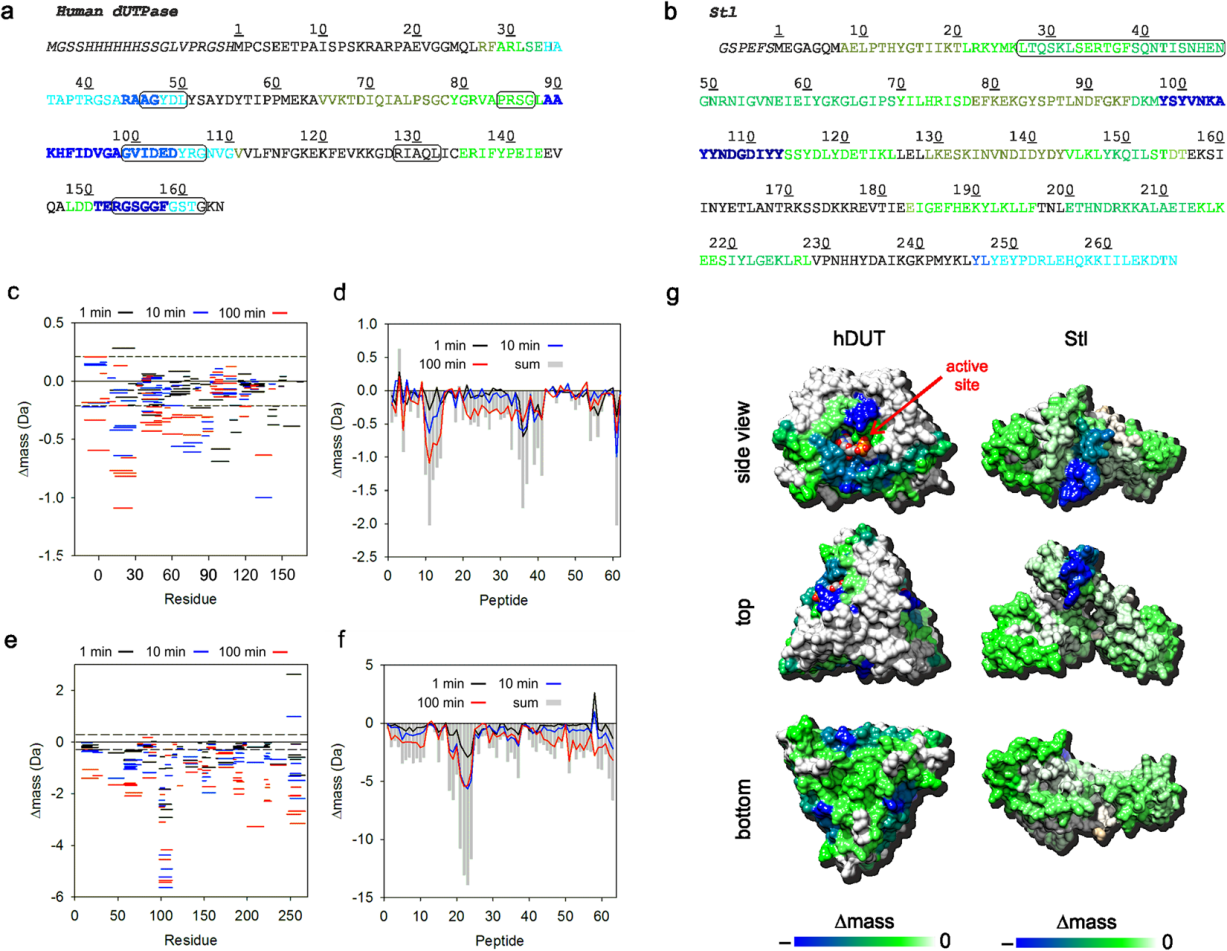
Representation of the hydrogen-deuterium exchange mass spectrometry results. (**A**,**B**) Sequence of human dUTPase and Stl proteins, respectively. Numbering starts at the first residue of the Uniprot sequences of the proteins (Uniprot IDs: P33316-2 and Q9F0J8 respectively). Extension compared to Uniprot sequence is in italics. Active site residues in case of dUTPase and the DNA binding motif of Stl are boxed. Sequence is colored according to HDX data (Δmass accumulated across all labelling times) applying color-scheme displayed on Panel G. (**C**–**F**) HDX-MS difference data (**C** and **E**) and associate Woods plots (**D** and **F**) for hDUT (**C**,**D**) and Stl (**E**,**F**) showing the change in isotope uptake upon complexation of the proteins. Labelling time points are indicated by different colors and the dashed lines in Panels C and E represent the 95% confidence bands. (**G**) Representation of the HDX-MS difference data on the surface of the human dUTPase and Stl. In case of the human dUTPase an apo state structure is shown (PDB ID: 1Q5U), the C-terminal 13 residues are omitted from the representation since the position of these residues were not resolved in the crystal structure presumably because of flexibility. Position of the substrate analogue is shown based on the structural alignment of the apo and ligand-bound structures (3EHW). The substrate analogue is shown as spheres with elemental coloring (carbon white, nitrogen blue, oxygen red, phosphorus orange) to visualize the interference of Stl and substrate binding. In case of Stl a Phyre2 generated model is shown, which was compatible with synchrotron radiation circular dichroism and mutagenesis results obtained for the protein. Coloring is according to the scale at the bottom of the panel.

The human dUTPase:Stl interaction was investigated by hydrogen-deuterium exchange mass spectrometry (HDX-MS). HDX-MS is a powerful biophysical technique that reports on time-dependent changes in the local deuterium uptake of a protein in D_2_O solvent^44^. The isotope uptake rates are dependent on the environment of exchangeable sites providing structural and dynamical information on these protein regions. The isotope uptake of the individual proteins as well as the premixed complex were characterized using a continuous labeling strategy recorded at three labelling time points (cf. Methods). The resulting HDX-MS patterns recorded for the complex were then subtracted from those obtained for the individual proteins to yield difference plots for both proteins which provide insight into the change in isotope uptake between the individual and assembled proteins (Fig. 2). The sequence coverage excluding tags for Stl and hDUT was 94.0% and 95.7%, respectively and the redundancy was over 2 in both cases (Supplementary Fig. 2).

The difference plot generated from the HDX-MS results of human dUTPase in the presence of Stl shows a clear negative mass shift that localizes to specific peptide regions (Fig. 2c, peptide numbering is shown on Supplementary Fig. 2). In order to understand these changes at the residue level, Woods plots were prepared from the HDX-MS difference outputs where the mass shift of each peptide is reported on the primary sequence of the protein (See methods)^69^. The most significant mass shifts of hDUT in the presence of Stl maps to a segment of residues 34H–50L whereas large mass shifts are also observed at the C-terminus of hDUT although these Δmasses converge more rapidly implying a weaker interaction in this region (Fig. 2c,d). Peptides 30–41 show modest but persistent changes implying the role of residues 89A–110G in the interaction.

The HDX-MS difference plots for Stl in the presence of human dUTPase shows a clear negative mass shift that localizes to a narrow band of peptides (Fig. 2e,f). When projected onto the Stl sequence the most significant mass shifts are localized for peptides 21–24 corresponding to a segment of residues 98Y–113Y (Fig. 2e,f). In addition we observed minor changes in isotope uptake of Stl across the entire length of the protein upon binding to dUTPase. This suggests that Stl may undergo a global decrease in dynamics in the bound state and that although the binding is localized to a specific region the interaction is communicated across the entire protein.

### SAXS analysis of Stl and human dUTPase complexes

**Figure 3:**
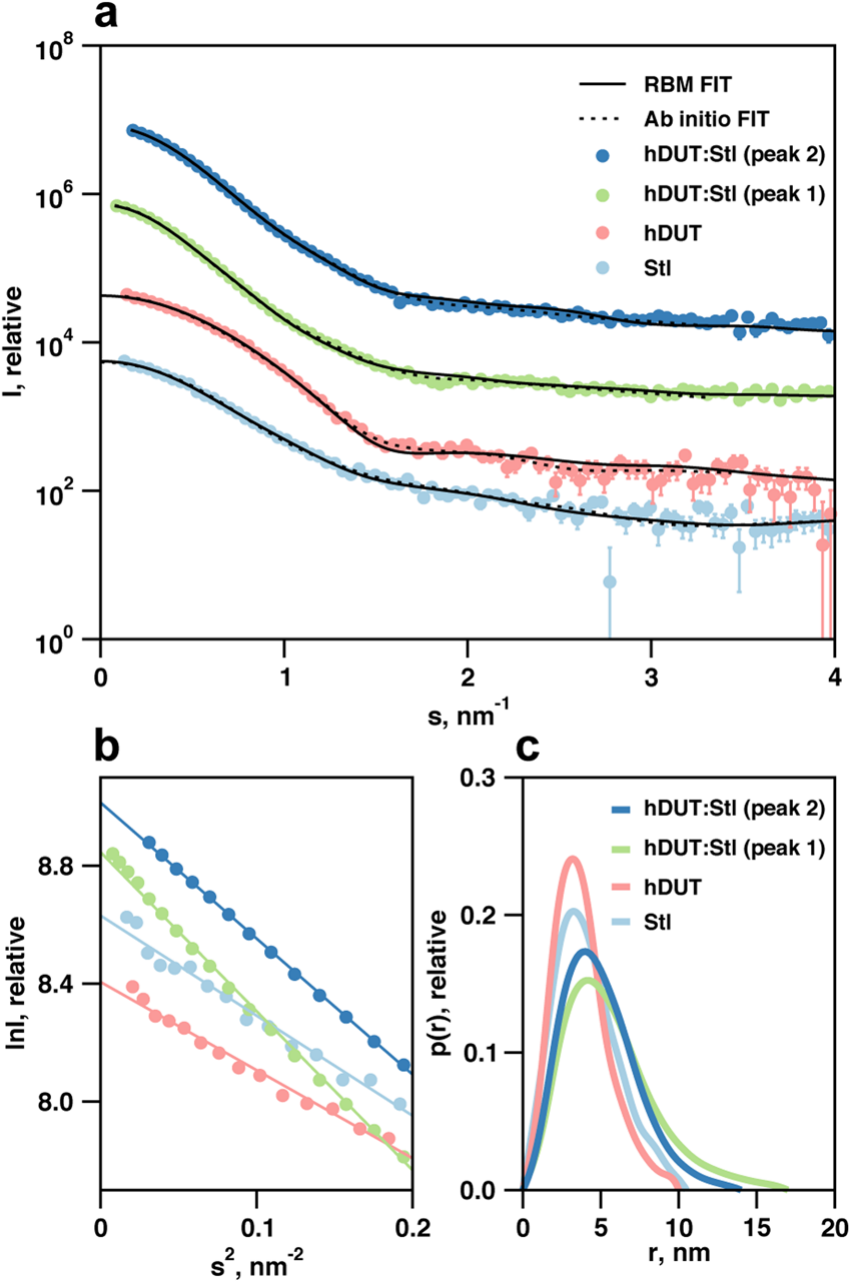
SAXS data for hDUT, Stl and complexes. (**A**) SAXS data (circles) and model fits (*ab initio*–dotted lines, rigid body models/RBM–solid lines) for human dUTPase (hDUT), Stl and hDUT:Stl complexes. The hDUT:Stl data shown corresponds to SEC-SAXS peak region 1 (green circles) and peak region 2 (dark blue circles). (**B**) Guinier plots of data in Panel A showing linearity of data at low angles, (**C**) Real-space pair distance distribution functions corresponding to an indirect Fourier transformation of the SAXS data in Panel A, showing the clear increase in maximum particle dimension upon interaction between the human dUTPase and Stl.

**Figure 4:**
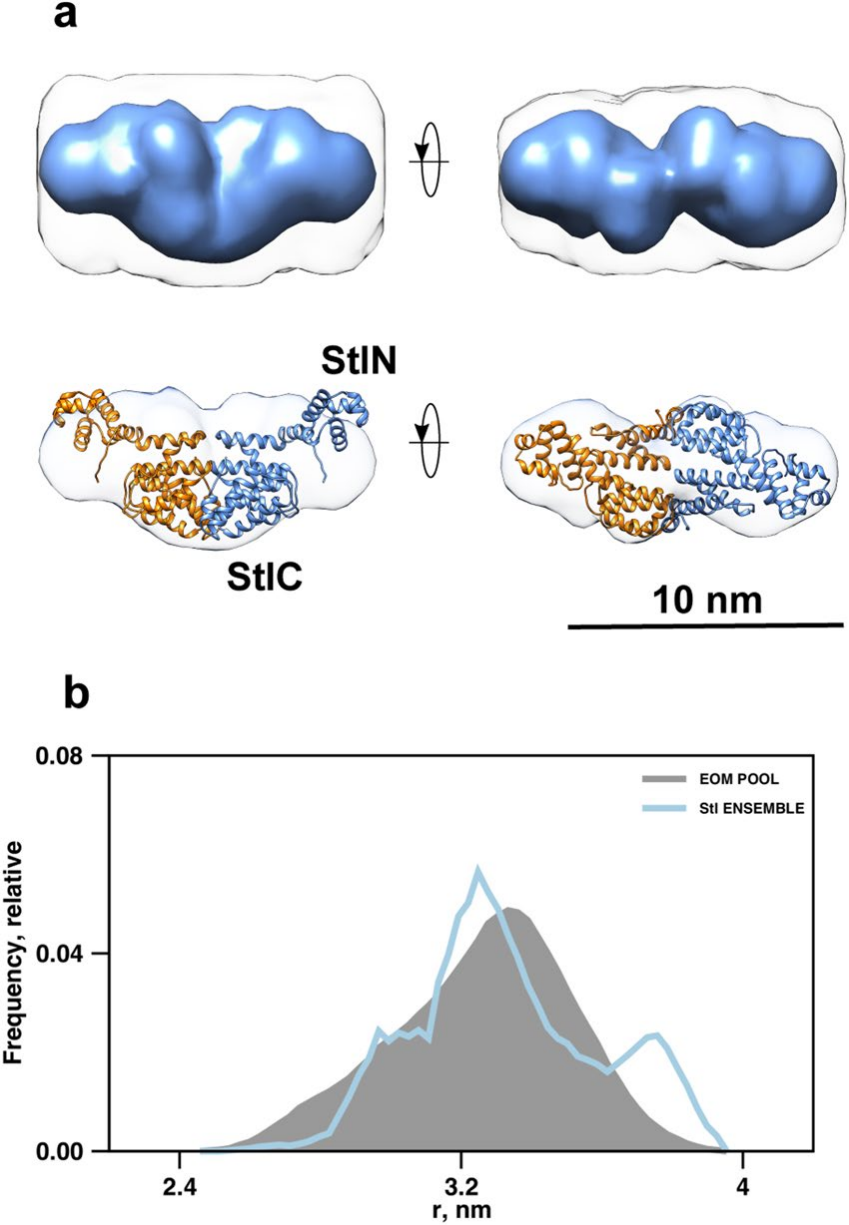
Models generated based on SAXS measurements for Stl. (**A**) Overlay of the dimeric Stl model from the Phyre2 server and the *ab initio* shape envelope reconstructed from SAXS data. StlN designates the amino terminal segment while StlC denotes the carboxy terminal segment of Stl according to ref.^67^. (**B**) R_g_ size-distribution from ensemble analysis of Stl SAXS data with EOM, showing the high level of flexibility of the selected ensemble relative to that of the random pool. The flexibility metric R_flex_ is 88% for both the selected ensemble and the initial pool, reflecting the presence of inter-domain flexibility while maintaining the dimeric interface.

**Figure 5:**
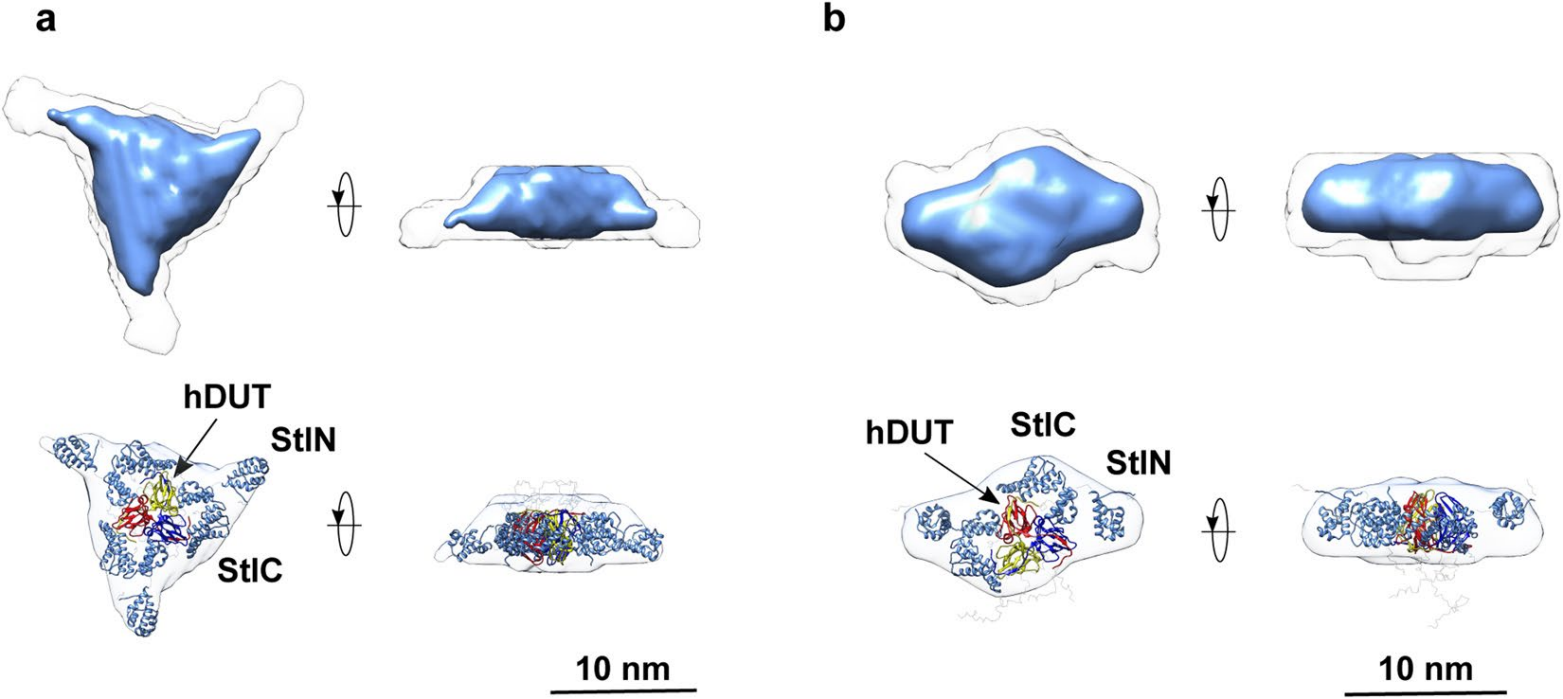
Models generated based on SEC-SAXS measurements for hDUT:Stl complex. Overlay of the SAXS/HDX-MS hybrid models of the hDUT_3_Stl_3_ (**A**) and hDUT_3_Stl_2_ (**B**) complexes and the *ab initio* shape envelopes reconstructed from SAXS data. The hDUT monomers are shown as red, yellow and blue cartoons. Stl monomers are shown as light-blue cartoons. Regions of missing sequence added during modeling as dummy residues are shown in wire format.

To assess the molecular shapes of hDUT and Stl and to determine the solution structure of the hDUT:Stl complex at low-resolution, SAXS experiments were performed. Results obtained are represented in Figs 3–5 and Tables 1 and 2. Previous SAXS studies performed on full-length hDUT demonstrate that the protein adopts a trimeric arrangement in solution consistent with the crystal structure (PDB ID: 1Q5U)^62,67^. Data obtained for hDUT yielded structural parameters and shapes consistent with single trimeric hDUT (Table 1). SAXS data for Stl suggested that the protein predominantly forms dimers in solution (Fig. 4 and Table 1), which is consistent with the dimer formation observed by native mass spectrometry and chemical crosslinking^42^ (Supplementary Figs 1 and 3). The *ab initio* models of the Stl dimer have an effective resolution of 4.9 ± 0.4 nm and average NSD of 1.5 ± 0.4. As no 3D structural information currently exists for Stl, structural models were generated using the Phyre2 server, validated by synchrotron radiation circular dichroism measurements^70^ and screened against the experimental SAXS data. A dimeric assembly of the best Phyre2 model was found to provide an excellent fit (χ^2^ = 1.0 between the model and experimental data) (Fig. 3a), which indicates that this is a valid model of the average structure in solution. This dimeric model was subsequently used for SAXS modeling procedures.

The observed relatively low resolution of the *ab initio* models for the Stl dimer suggests flexible nature of the protein. Thus the flexibility of the Stl dimer was investigated using an ensemble based approach, where based on chemical crosslinking results (Supplementary Fig. 3) the dimer interface was formed by the C-terminal region (T87-N267) of the Stl model and was fixed while random configurations of the N-terminal region (M1-K83) were generated. The results of this procedure are represented in size-distributions calculated from the pool and the selected ensemble of best-fitting configurations, with both distributions appearing to be similarly broad thus supporting a highly flexible domain arrangement of Stl protein (Fig. 4b). Domain flexibility in a dynamic system involving Stl-DNA interactions and modulation of that by Stl:hDUT complexation may be functionally important, so this was taken into account for subsequent hybrid modelling procedures.

To evaluate the formation of hDUT:Stl complexes and determine probable stoichiometry and three-dimensional structures, in-line SEC-SAXS experiments were conducted. A single peak with a small leading edge was observed (Supplementary Fig. 4), and individual scattering frames were examined across this peak. The SAXS parameters extracted from these frames show two self-consistent regions, with correlation map analysis^71^ identifying two pools of similar frames referred to as region 1 and region 2 (Table 2, Supplementary Fig. 4). The molecular weight parameters of region 1 and region 2 are consistent with 3:3 and 3:2 hDUT:Stl complexes, respectively, which were expected based on ITC and ESI-MS data. The existence of two type of complexes is also suggested by the higher *R*_*g*_ and *D*_*max*_ of region 1 compared to region 2 (45 Å and 170 Å *vs* 39 Å and 140 Å, respectively). The real-space distance distribution functions, *P*(*r*) for both SEC-SAXS regions are characteristic of extended structures, with the region 1 data clearly more extended than that of region 2 (Fig. 3c). *Ab initio* shape reconstructions for the region 1 and region 2 peak data yielded models that are clearly consistent with that expected for heterohexameric and heteropentameric hDUT:Stl assemblies (Fig. 5). The effective resolution is estimated at 3.7 ± 0.3 nm and 5.3 ± 0.4 nm for the 3:3 and 3:2 models, respectively.

Both reconstructions can easily accommodate a trimeric hDUT core with the remaining volume occupied by three or two Stl monomers, respectively. Interestingly, no reasonable solutions for the complexes could be obtained using a dimeric Stl model, suggesting that disruption of the Stl dimer is inevitable for the formation of this complex. Encouraged by the *ab initio* modeling results, rigid body structure calculations were conducted using the SAXS data and constraints based on HDX-MS using the trimeric hDUT core and Stl monomers taken from the Phyre2 homology model. The top 20 models obtained for hDUT_3_Stl_3_ were clustered based on the relative position of the N-terminal (residues 1–84, StlN) and C-terminal (residues 85–267, StlC) segments of Stl to the dUTPase substrate binding pocket (Supplementary Table S1). HDX-MS data shown here and our former experiments^70^ showed that StlC directly interacts the dUTPase, while StlN has a limited contribution. It is important to note that the models showing the best χ^2^ statistics were also the ones satisfying these criteria (cf. models 11 and 17, in Cluster 1 in Supplementary Table S1). A representative model is shown on Fig. 5 (model 11, χ^2^ = 1.4, Fig. 3a). The best model hDUT_3_Stl_2_ matching with this quaternary structure (Fig. 5) had also a good agreement with the SAXS data (χ^2^ = 1.2, Fig. 3a). These models also correspond well with the HDX-MS results as most of the dUTPase and Stl surface which showed negative HDX-MS signal, is buried in the complex (cf. Supplementary Fig. 5 and 6). Note that the HDX-MS results are referring to data obtained for tryptic peptide fragments, so it is possible that not all residues in a peptide are uniformly responsible for the signal, and involved in protein-protein interaction. Although more detailed information about the role of individual residues could only be assessed by extensive mutagenesis study of this complex, which is beyond the scope of this study.

## Discussion

Previous studies have provided evidence that the staphylococcal repressor protein Stl can form a complex with the trimeric dUTPases from *S*. *aureus Φ*11 and 80*α* phages, *M*. *tuberculosis*, and *D. melanogaster in vitro*^21,42,43^, although the latter two proteins share only modest sequence similarity with the original phage dUTPase partners of Stl. Using various techniques we have now demonstrated that Stl also interacts with the human dUTPase, which displays 62% sequence similarity to the phage dUTPases (Supplementary Fig. 7). Table 3 shows the data available to date on complexation of Stl and dUTPases from various sources.

**Table 3:** Summary of the results on dUTPase-Stl interaction.

Origin of dUTPase		Origin of dUTPase	Characteristics of interaction		*In vivo* functional effect
			Affinity (K_d_*)	dUTPase inhibition	
*S*. *aureus* phages	* Φ11*	β-pleated trimer	1.84 nM (BLI^77^), 100 nM (ITC^42^) 62 nM (QCM^42^)	K_i,app_ = 27 nM^42^ K_i,app_ = 1.2 nM^78^ ca. 100% inhibition^42,78^	induction of SaPI_bov1_ transfer in *S*. *aureus*^40^
	*80α*	β-pleated trimer	40 nM (BLI^77^)	n.d.^#^	induction of SaPI_bov1_ transfer in *S*. *aureus*^40^
	*φNM1*	all-α helical dimer	qualitatively verified by Ni-NTA co-elution, crosslink SDS-PAGE, EMSA^39^	activity decrease in the presence of Stl^39^	induction of SaPI_bov1_ transfer in *S*. *aureus*^39^
prokaryote	*M*. *tuberculosis*	β-pleated trimer	qualitatively verified by native gel electrophoresis^21^ EMSA^78^	max. 84% inhibition K_i,app_ = 5.5 nM^21^	Stl expression: perturbed colony formation and increased cellular dUTP level in *M*. *smegmatis*^21^
insect	*Drosphila melanoglaster*	β-pleated trimer	n.d.^#^	max. 40% inhibition K_i,app_ = 30 nM^43^	n.d.^#^
mammal	human	β-pleated trimer	230 nM (ITC)^$^	max. 70% inhibition^$^ K_i,app_ = 6.7 nM^$^	n.d.^#^

*Where available. ^#^Non-defined. ^$^This work.

We have shown that the Stl-dUTPase interaction has strong functional consequences with regard to the biological function of both proteins in the complex (cf. Table 3). Namely, complexation inhibits both i) enzymatic activity of dUTPase, and ii) DNA-binding capability of the Stl repressor.

To understand the mechanism of these dual inhibitory effects, detailed biophysical and structural information about the complex is indispensable. Here we exhibit such hitherto unseen insights applying cross platform approach, by combining ITC, mass spectrometry, SEC-SAXS and HDX-MS results to analyze the complex of human dUTPase with Stl. Our results presented herein argue altogether for the formation of hDUT_3_Stl_2_ and hDUT_3_Stl_3_ complexes. The 3:2 stoichiometry was also suggested for the complex of *Φ11* phage and *D*. *melanogaster* dUTPases with Stl based on native MS^42,43^. It is important also to note that a higher order complex with Stl was suggested to exist for *Φ11* phage dUTPase based on native gel electrophoresis results^42^.

Kinetic analysis revealed a slow and tight binding interaction between Stl and Φ11DUT where Stl and dUTP competes for the active site of the dUTPase^42^. We observed drastic dependence of human dUTPase inhibition and complex formation on the order of mixing of components in case of the kinetic studies and the native gel electrophoresis. Since we only observed inhibition or complex formation when Stl was premixed with hDUT, we suggest that Stl potentially acts through a slow and tight binding mechanism on the human dUTPase, similarly to that of Φ11DUT^42^. The Stl protein and the substrate dUTP were shown to compete with each other for dUTPase binding in case of Φ11DUT^42^. Our native gel result argues for similar mechanism in case of human dUTPase. Although, the maximal inhibitory effect of Stl exerted on Φ11DUT and hDUT differs (ca.100 vs 70% inhibition, respectively), these two dUTPases seem to accommodate Stl in similar ways. However the differences that result in the species specific inhibitory effect remains to be explored. In addition to the trimeric dUTPases, Stl also inhibits the dUTPase of the φNM1 phage^39^ although this enzyme belongs to the dimeric dUTPase family associated with a completely different all-α-helical fold as compared to the beta-pleated trimeric enzymes. It is plausible to suggest that the dUTP binding pocket is directly involved in complex formation based on the observed general inhibitory effect of Stl on enzymatic activity on differently folded dUTPases.

To reveal the interaction surface of the human dUTPase with Stl, hydrogen deuterium exchange mass spectrometry measurements were performed on the individual proteins and the complex. Overall the HDX-MS data provide additional and conclusive evidence of a human dUTPase:Stl interaction, moreover these data also reveal that the interaction is localized to specific regions of the proteins. In the case of the human dUTPase the most significant changes were observed for peptides from the regions covering residues 34H–50L and 89A–110G, which overlap with the first three conserved motifs of dUTPases (Fig. 2a). In addition to these segments, H-D exchange rates at the carboxy terminal region of the human dUTPase, which includes the fifth conserved motif, was also affected by the complex formation. However, fluctuations of the H-D exchange rates point to a weaker and transient interaction involving this segment. The three active sites within trimeric dUTPases are all built from residues of the five conserved motifs from all the 3 subunits in a specific pattern. Namely, each active site is constituted as follows: one subunit donates conserved motifs 1, 2 and 4 and another contributes motif 3 to form the substrate binding cavity between two subunits, which is closed by the flexible motif 5 of the third protomer upon substrate binding. This closed conformation of motif 5 creates the catalytically competent active site architecture by providing conserved residues with decisive role in enzyme catalysis^72–74^. It is apparent that the interaction surface of human dUTPase with Stl identified by HDX-MS directly involves the substrate binding pocket (Fig. 2g). We also showed that dUTP and Stl binding to dUTPase are mutually exclusive (Fig. 1d and E)^21,42^, so that Stl can only bind to the substrate-free form of dUTPase. In this substrate-free conformation, the conserved motif 5 is flexible and the active site is accessible^62,67,75,76^.

Taken together the HDX-MS and enzyme kinetic data, we suggest a mechanistic model in that Stl is allowed to dock into the substrate binding cavity of dUTPase only if access to the cavity is not hindered by either the substrate or the closed conformation of the flexible motif 5. The complex may be further stabilized by interactions involving the flexible motif 5 of the human dUTPase and Stl, which is consistent with the observed decrease in the H/D exchange rate of the conserved motif 5 upon complex formation. However, this potential additional effect possesses transient characteristics according to the HDX-MS data, which is consonant with our previous results, that Stl binding was not perturbed in the case of the Φ11DUT mutant lacking motif 5^42^. Although based on mutational analysis the contribution of this segment to the protein-protein interaction varies between 80α and Φ11 dUTPases, the experimental data for interaction of phage dUTPases with Stl is also in agreement with our model^77^. It is also worth to note that a region including the β-and γ-phosphate coordinating motif 2 showed significant decrease in the H/D exchange rate, which is consistent with the previous finding that only dUTP but not dUMP perturb the complex formation of dUTPases with Stl^42,77^.

In the case of Stl, the HDX-MS measurements revealed that a specific Tyr-rich region of the Stl protein (segment 98Y–113Y) showed significant H/D exchange rate decrease upon addition of dUTPase. This result implies that this segment became less solvent accessible, which indicates that it is involved in the interaction with dUTPase (Fig. 2e,f). This finding is consistent with our previous results that removal of the N-terminal segment (residues 1–85) of Stl did not perturb the protein-protein interaction^70^. The global negative HDX data observed for the peptides along the whole Stl sequence might correspond to the decrease in the flexibility of the protein upon Stl-dUTPase complex formation. We have not observed significant positive HDX signal, which would presumably be a consequence of the dissociation of the Stl dimer. These experimental data together with previous results on dUTPase-Stl interactions reinforce our postulation that the dimer interface of Stl might overlap with the Stl-hDUT interaction surface^70^.

**Figure 6:**
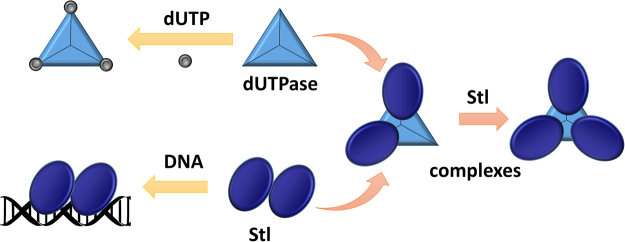
Schematic model of hDUT:Stl complex assembly and Stl-DNA interaction. According to our results human dUTPase is not able bind to Stl and its substrate simultaneously, and is inhibited by Stl. Stl-DNA complex is also disrupted by hDUT. We showed experimental evidence on the existence of hDUT_3_Stl_3_ and hDUT_3_Stl_2_ complexes. The 3D structural models for hDUT:Stl complexes presented here revealed that interaction of trimeric dUTPase with Stl leads to disruption of the Stl dimers.

This previous hypothesis is well supported here by the 3D models of the hDUT_3_Stl_3_ and hDUT_3_Stl_2_ complexes generated from the experimental SEC-SAXS data applying HDX-MS results as restraints (cf. Fig. 5). Optimal solutions were not obtained in hybrid SAXS modeling with HDX-MS restraints if dimeric Stl was used. Indeed, the only models providing excellent agreement with the experimental data were those generated from trimeric hDUT and two/three Stl monomers (cf. Fig. 3a and Supplementary Figs 5 and 6). Recently developed strategies to simulate HDX-MS difference data for quantitative scoring of docking outputs, may stimulate further refinement studies beyond the scope of this work^69^. This pioneering 3D model provides an insightful explanation for the dUTPase-induced perturbation of DNA-binding by Stl, since Stl is expected to exert its repressor function as dimer. We suggest that Stl binding to trimeric dUTPase initiates dissociation of the Stl dimer with concomitant perturbation of the Stl-DNA interaction (cf. Fig. 6). Strikingly, a somewhat analogous mechanism was suggested for the Stl-dimeric dUTPase interaction, however, in that model both Stl and dUTPase oligomers fall apart to form a heterodimer of one Stl and one dUTPase monomer^39^. It seems that Stl-dUTPase complexation has some general characteristics but also show unique traits specific to the actual dUTPase partner (cf. also Table 3). Our focused study on the structural requirements of human dUTPase / Stl interaction may therefore serve as the starting point of future development of a species-specific dUTPase inhibitory peptide or protein.

## Conclusions

The human dUTPase has a prominent role in guarding genome integrity via removal of dUTP from the nucleotide pool, which designates this enzyme as a target for onco-therapies. Accordingly, small molecular drug-candidates were developed to inhibit dUTPase function and some of these are currently evaluated in Phase 1 trials^33^. Here we describe the discovery that the staphylococcal Stl repressor is a potent protein inhibitor of human dUTPase and as such, it can be used as a versatile tool to decipher the cellular pathways involving dUTPase function. Detailed understanding of the molecular mechanism of action of complex formation between human dUTPase and Stl, as well as the ensuing functional effects requires in-depth biophysical characterization.

The present results on complexation of human dUTPase:Stl and the obtained structural model based on HDX-MS together with an integrated structural biology approach and complex structure provide plausible explanations for mutual inhibition of Stl and dUTPase physiological function in their complex. On one hand, HDX results clearly delineated peptide segments around the dUTPase active site that are involved in binding to Stl and these data are in line with the observed inhibition of the dUTPase enzymatic function and competition between Stl and dUTP for binding to dUTPase. Importantly, the entrance to the dUTP accommodating beta-hairpin (ie conserved Motif 3) as well as the interaction surface for beta-gamma phosphate-chain of the substrate (ie conserved Motif 2) are both identified in our present study as involved in Stl binding. On the other hand, loss of the DNA-binding capability of Stl in its complex with human dUTPase is rationalized in light of the model resulting from SEC-SAXS measurements that necessarily involves Stl monomers hereby disrupting the functional repressor homodimer.

We conclude that proteinaceous inhibition of human dUTPase by Stl offers a novel, promising tool to investigate dUTPase function in different systems and propose further exploitation of Stl as a dUTPase-specific inhibitor. Our evidence-based structural model offers unparalleled insights into the mechanism of Stl-dUTPase complexation in general. Additionally, the model clearly delineates the peptide segments of Stl involved in interaction of the human enzyme alluding to the possibility of development of peptide based inhibitors. Since dUTPase is a key factor in genome integrity, the potentials of Stl or its derivatives to be used as a specific inhibitor cannot be underestimated.

### Data Availability Statement

SAXS data is deposited in the Small Angle Scattering Biological Data Bank (www.sasbdb.org), with accession codes: SASDC57, SASDC67, SASDC77, SASDC87.

All other data generated or analysed during this study are included in this published article (and its supplementary information files).

## Electronic supplementary material

Supporting Information
