# Supplementary material for: Structural model of human dUTPase in complex with a novel proteinaceous inhibitor
Source: Sci Rep. 2018 Mar 12;8:4326. doi: 10.1038/s41598-018-22145-8 (PMC5847570; doi:10.1038/s41598-018-22145-8)

Supporting Information

Structural model of human dUTPase in complex with a novel proteinaceous inhibitor

Kinga Nyíri[1,2,*], Haydyn D.T. Mertens[3], Borbála Tihanyi[2], Gergely N. Nagy[1,2 †], Bianka Kőhegyi[1], Judit Matejka[1], Matthew J. Harris[4], Judit E. Szabó[1,2], Veronika Papp-Kádár[1,2], Veronika Németh-Pongrácz[2], Olivér Ozohanics[5], Károly Vékey[5], Dmitri I. Svergun[3], Antoni J. Borysik[4], Beáta G. Vértessy[1,2,*]

[1] Department of Applied Biotechnology and Food Sciences, Budapest University of Technology and Economics, Budapest, 1111, Hungary

[2] Institute of Enzymology, Research Centre for Natural Sciences, Hungarian Academy of Sciences, Budapest, 1117, Hungary

[3] European Molecular Biology Laboratory, Hamburg Outstation, c/o DESY, Notkestrasse 85, Hamburg 22603, Germany

[4] Department of Chemistry, King's College London, Britannia House, London SE1 1DB, United Kingdom

[5] Institute of Organic Chemistry, Research Centre for Natural Sciences, Hungarian Academy of Sciences, Budapest, 1117, Hungary

[*] To whom correspondence should be addressed. Tel:+36 1 382 6707; Fax: +36 1 463 3854;
Email: vertessy@mail.bme.hu, vertessy.beata@ttk.mta.hu
Correspondence may also be addressed to Kinga Nyíri. Tel: +36 1 382 6729;
Email: nyiri.kinga@ttk.mta.hu

[†] Present address: Division of Structural Biology, University of Oxford, Roosevelt Drive, Oxford OX37BN, United Kingdom

Experimental Procedures

Mutagenesis

Stl was expressed from a derivative of pGEX-4T-1 vector described first in [1] encoding Stl protein with an N-terminal His-tag fused C-terminal to a thrombin cleavable GST-tag. The His-tag was removed using the mutagenesis primers 5'-GTGGATCCCCGGAATTCAGCATGGAAGGCGCG-3' and 5'-CGCGCCTTCC ATGCTGAATTCCGGGGATCCAC -3'. The resulting construct was validated by DNA sequencing at Eurofins MWG Operon. Amino acid sequence of the protein after cleavage of the GST-tag is presented on Figure 2 of the main text.

Purification of Stl

Purification of Stl was performed as described previously [1]. Briefly the pellet was resuspended in buffer A (50 mM HEPES/NaOH, pH=7.5, 200 mM NaCl) supplemented with ca. 2 μg/ml RNase and DNase

and a Complete ULTRA mini EDTA-free protease inhibitor tablet (Roche) and after 4 x 60 s sonication the suspension was centrifuged at 16000 g for 30 min. The supernatant was applied onto a benchtop glutathione-agarose affinity-chromatography column (GE Healthcare). After 30 min of equilibration, the matrix was washed with ten bed volumes of Buffer A. Then GST tag was cleaved during overnight (ca. 16 h) incubation at 18°C by 80 unit thrombin in 4 mL of buffer A applied onto the matrix and pure Stl protein was obtained in the flow-through.

Purification of human dUTPase (hDUT)

Purification of hDUT was performed as described in an earlier study [2]. Briefly, cells were resuspended in lysis buffer (50 mM TRIS·HCl, pH=8.0, 300 mM NaCl, 0.5 mM EDTA, 0.1% Triton X-100, 10 mM 2-mercaptoethanol, 5 mM benzamidin, 1 mM PMSF; ca. 2 µg/ml RNase and DNase and a Complete ULTRA mini EDTA-free protease inhibitor tablet (Roche)). After 4 x 1min sonication, the supernatant from centrifugation at 16.000 x g for 30 min was applied onto a Ni-NTA column (Novagen) pre-equilibrated with lysis buffer containing 15 mM imidazole. After removing the contaminants by washing the column with ten bed volumes of low salt and high salt buffers (50 mM HEPES pH=7.5, supplemented with 30 mM KCl or 300 mM KCl, respectively), His-hDUT was eluted with 500 mM imidazole dissolved in low salt buffer. After elution His-hDUT was dialyzed against buffer B (50 mM HEPES, pH=7.5, 300 mM NaCl, 5 mM $MgCl_2$). His-hDUT was gelfiltrated in buffer B on a S200 Increase 10/300 column. For the native mass spectrometry and the batch SAXS measurements tag-free hDUT was used. The His tag was removed from His-hDUT by adding 40 unit thrombin to 1 ml of 18 mg/ml His-hDUT and incubated overnight at 20°C, after cleavage the protein was purified by SEC as described above. His-hDUT was referred as hDUT throughout the main article.

Chemical crosslinking

Samples of full-length Stl and StlC – a truncated mutant containing the carboxy terminal segment of Stl (residues 85 – 267) of 40 µM concentration were incubated with 0.1 – 0.4 mM disuccinimidyl suberate (DSS) at 20°C for one hour. Quenching of the crosslinking reaction was performed by the addition of 5 µL 100 mM (pH 7.5) Tris buffer to 40 µL of samples and then those were analyzed by SDS-PAGE on a 12% gel using Page Ruler prestained protein ladder as a molecular weight marker. Gels were stained by Page Blue protein staining solution (Thermo Fisher).

Supplemental Figures

Figure S1

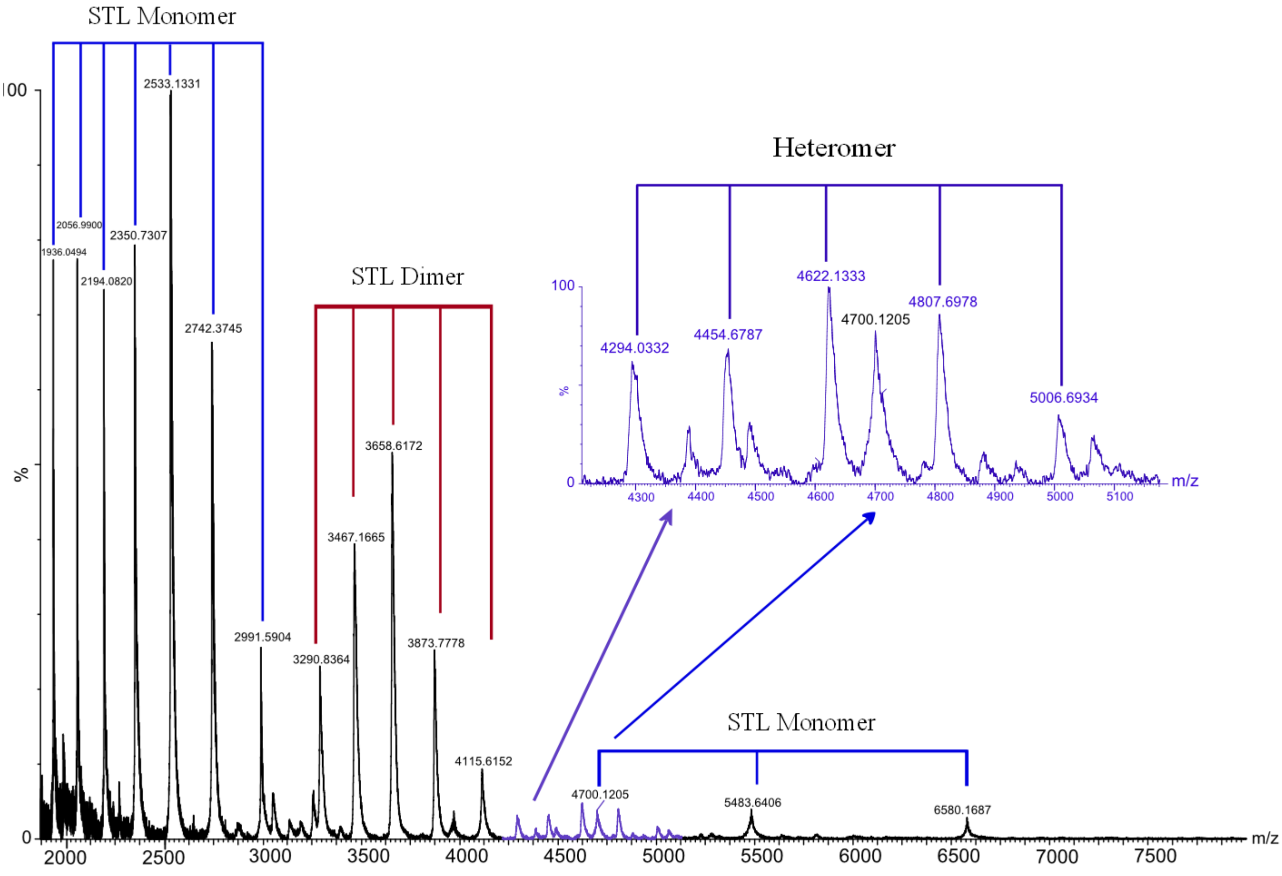

Figure S1. Annotated mass spectra of the mixture of the Stl and human dUTPase proteins measured under native electrospray conditions. Peaks corresponding to Stl dimer are marked with a red frame. In the inset the 4300-5100 m/z region of the spectra is magnified. The violet frame highlights the peaks corresponding to the heteromer with 120.0 ± 0.1 kDa molar weight, which argues for a $hDUT_3Stl_2$ complex, in which a human dUTPase trimer (54.1 kDa) binds to two Stl molecules (64.0 kDa).

Figure S2

a

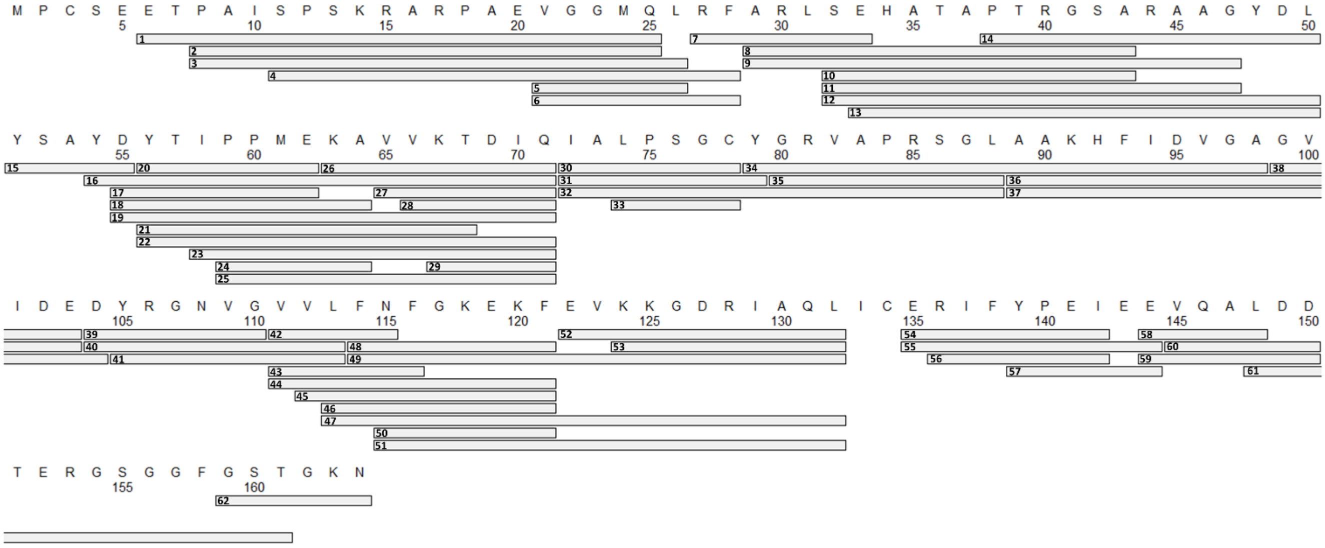

b

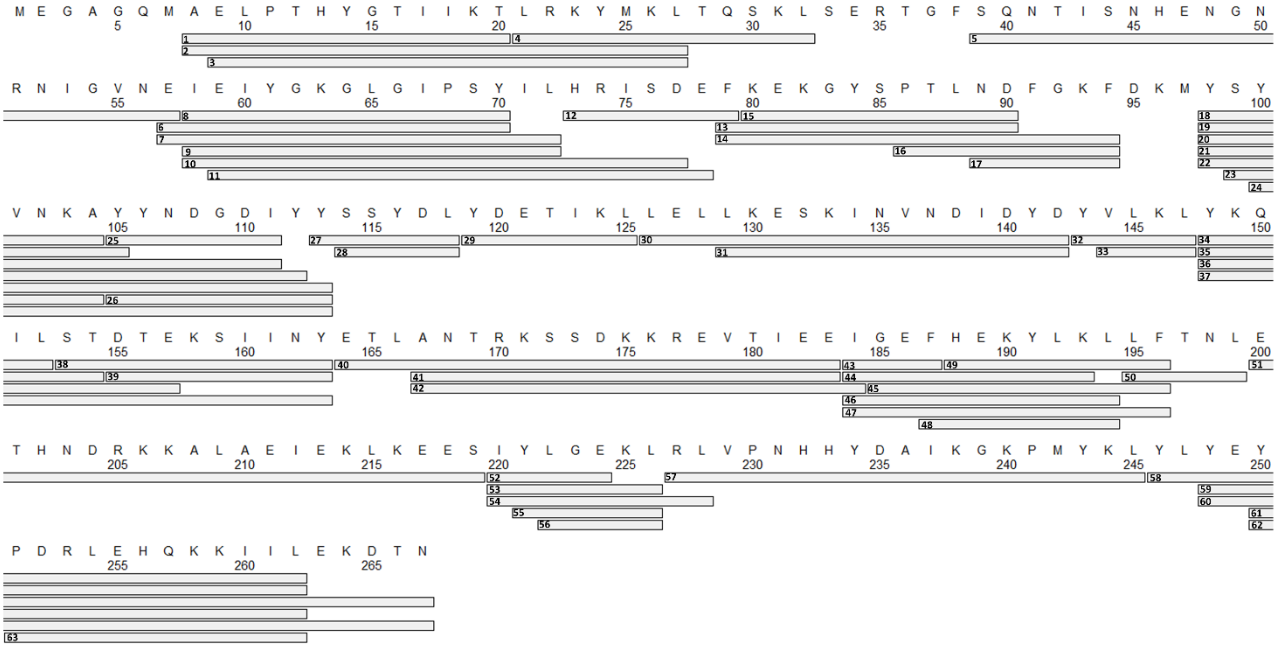

Figure S2. Peptides assigned on HDX-MS difference plots A) Coverage map of human dUTPase (hDUT) describing the distribution of 62 individual peptides (horizontal bars) covering 95.7% of the hDUT sequence. **B)** Coverage map of Stl describing the distribution of 63 individual peptides (horizontal bars) covering 94.0% of the Stl sequence. Numbering of peptides are included on both figures.

Protein (40 µM)	Stl		StlC			
DSS (mM)	–	0.2	–	0.1	0.2	0.3

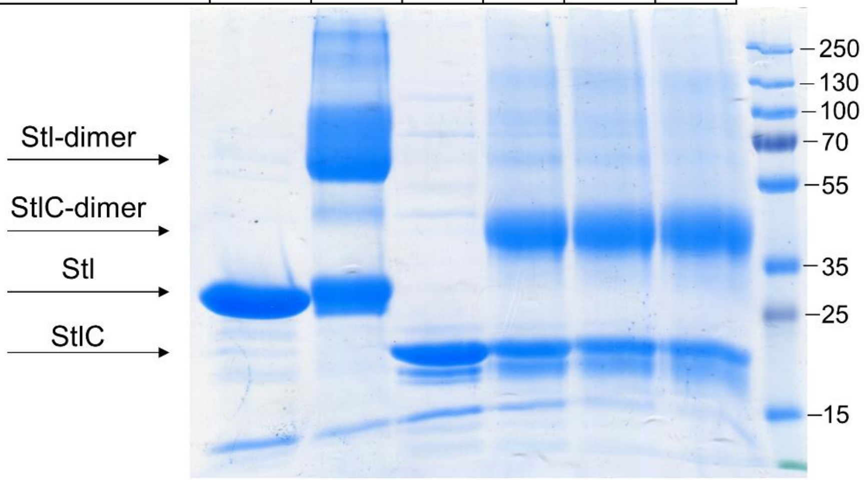

Figure S3. Chemical crosslinking of full-length Stl and StlC – a truncated mutant containing the carboxy terminal segment of Stl (residues 85 – 267). Upon addition of the crosslinking agent, DSS to Stl (Mw: 32 kDa) and StlC (Mw: 22.5 kDa) bands corresponding to the size of Stl and StlC dimers appeared.

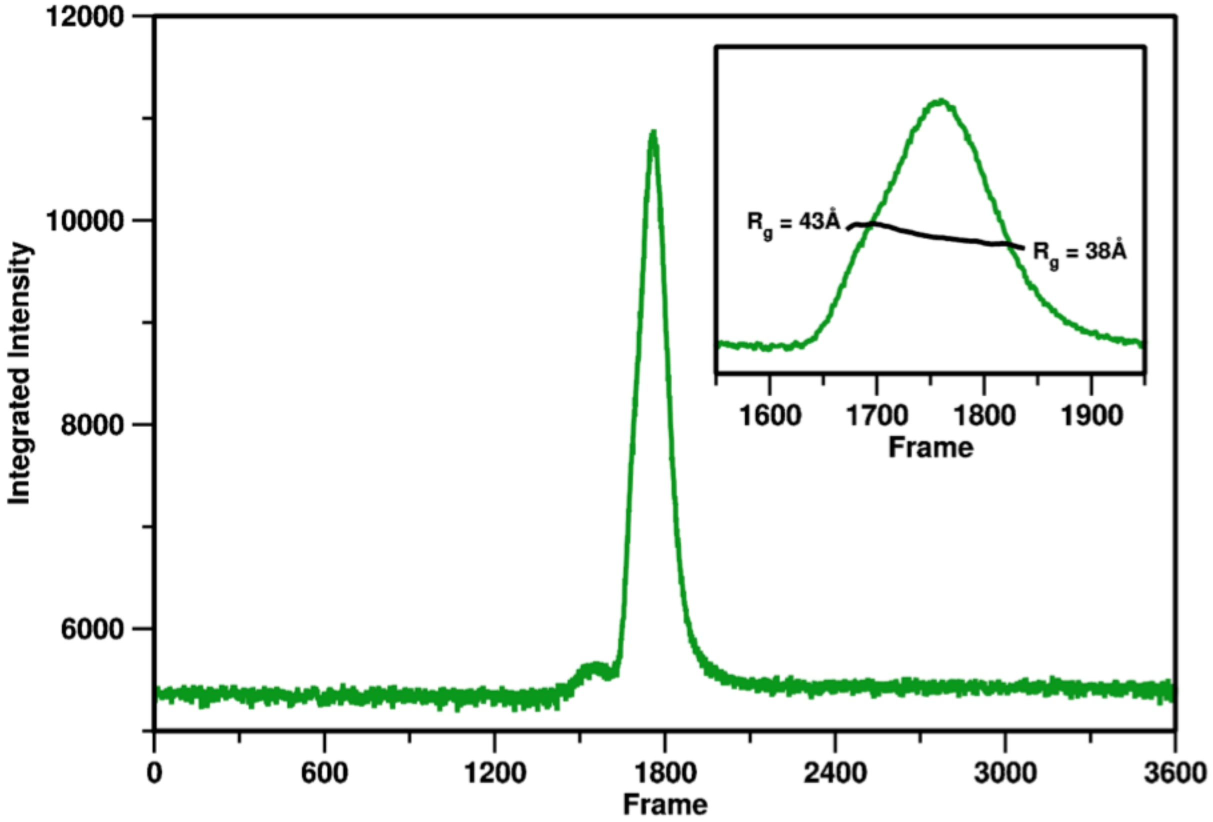

Figure S4. Chromatogram of SEC-SAXS measurement of the hDUT:Stl complexes. The integrated SAXS intensity vs frame number is shown. Protein was loaded at ~8 mg/ml on a Sepharose S200 Increase 10/300 column and run at 0.5 ml/min in 50 mM HEPES, pH=7.5, 300 mM NaCl, 5 mM $MgCl_2$. The inset shows a zoomed view of the major peak and the average radius of gyration computed from the data using a sliding window of 10 frames (black line).

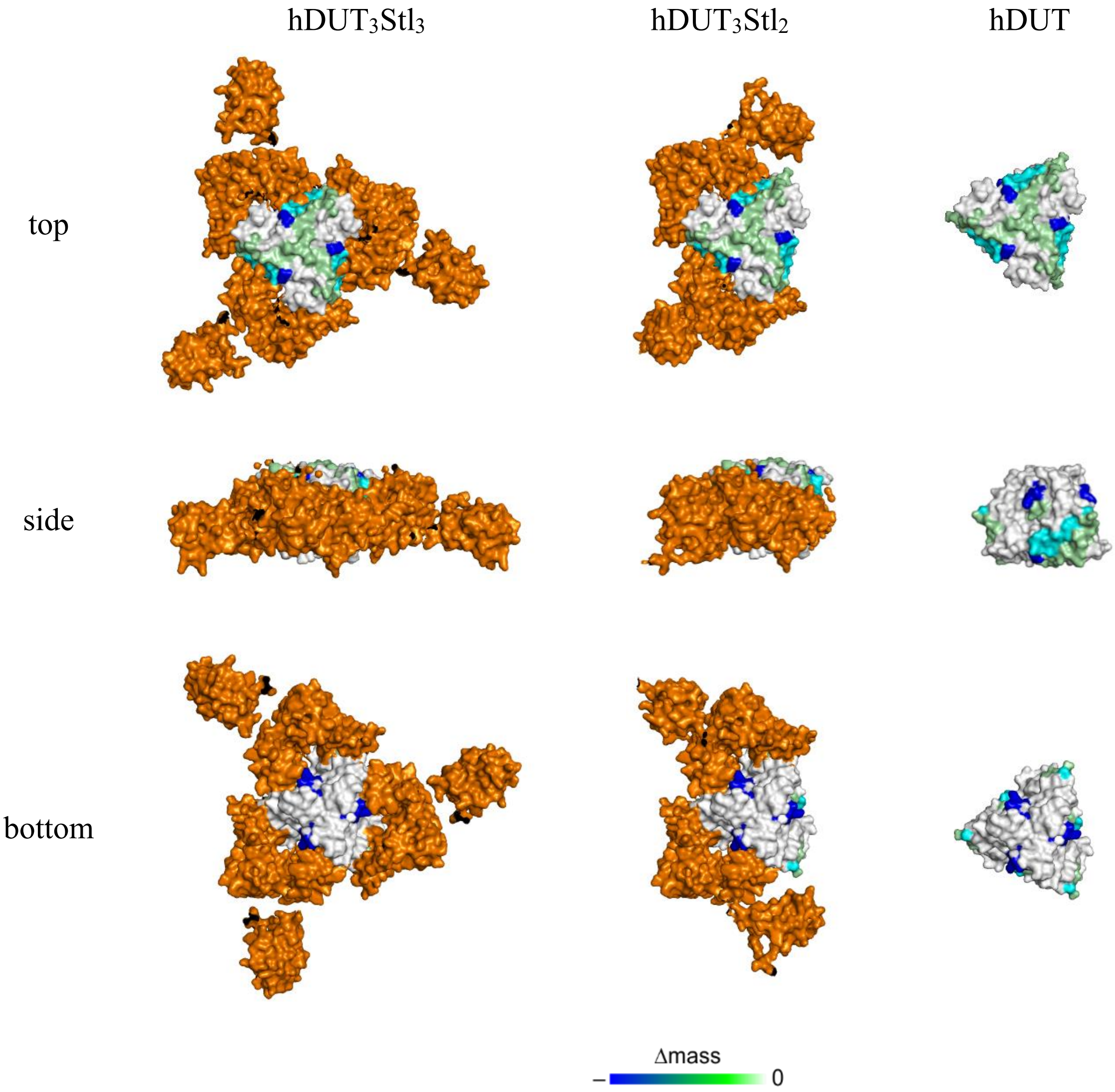

S5 Figure. Surface representation of Stl-dUTPase complexes. Stl is coloured orange, hDUT coloured according to HDX-MS applying the color-scheme displayed on the bottom of the figure. Most of the dUTPase surface which showed negative HDX MS signal is buried by Stl in the complex.

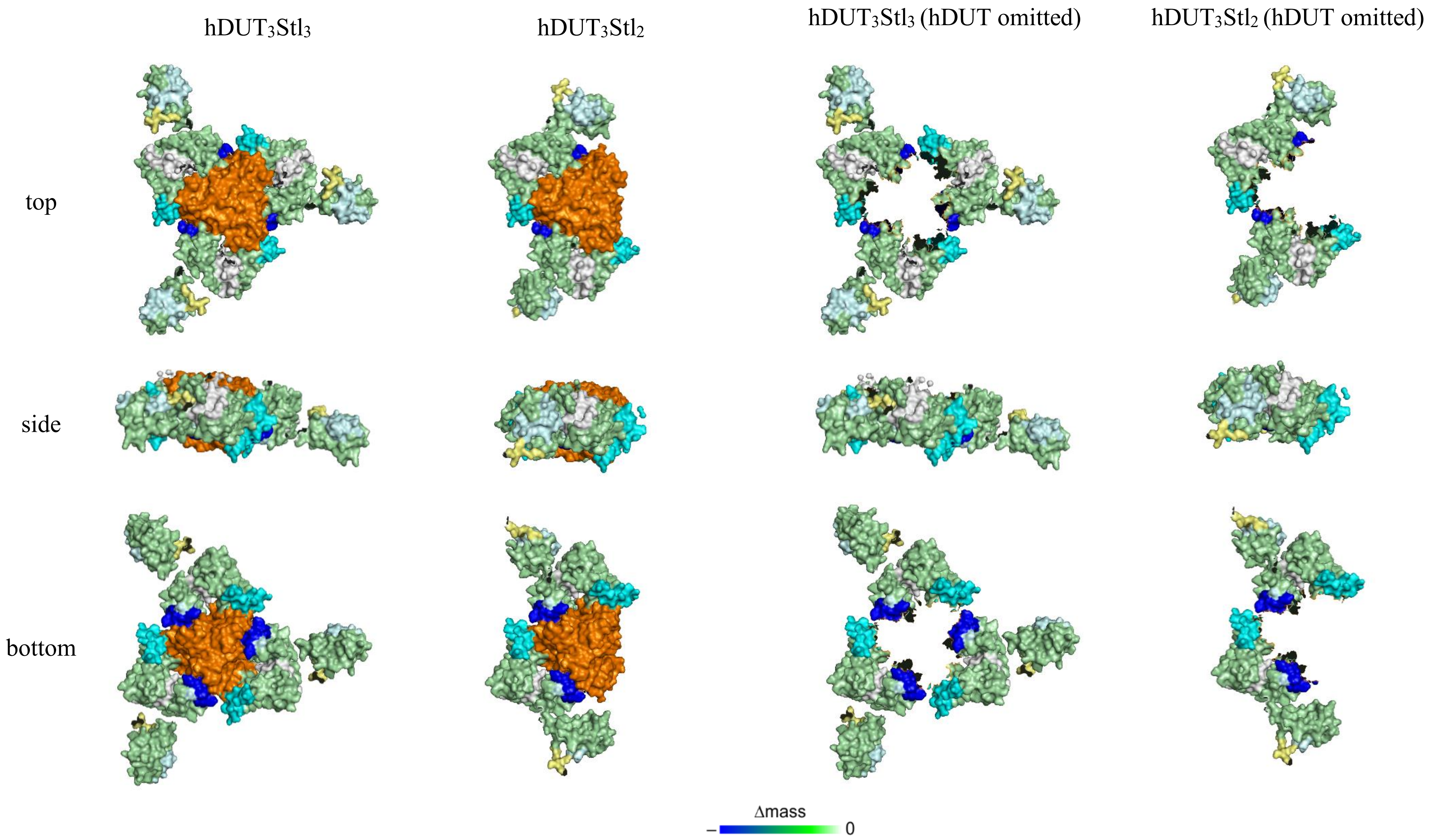

S6 Figure. Surface representation of Stl-dUTPase complexes. hDUT is coloured orange, Stl coloured according to HDX-MS applying the color-scheme displayed on the bottom of the figure. Most of the Stl surface which showed the most negative HDX-MS signal is buried by hDUT in the complex.

```
                        10        20        30        40        50        60
                         |         |         |         |         |         |
Φ11DUT          MTNTLQVRLLSENARMPERNHKTDAGYDIFSAETVVLEPQEKAVIKTDVAVSIPEGYVGL
hDUT            MQ--LRFARLSEHATAPTRGSARAAGYDLYSAYDYTIPPMEKAVVKTDIQIALPSGCYGR
                *   *:.  ***:*  * *.    ****::**   .: * ****:***: :::*.*  *

                        70        80        90       100       110       120
                         |         |         |         |         |         |
c11DUT          LTSRSGVSSKTHLVIETGKIDAGYHGNLGINIKNDAIASNGYITPGVFDIKGEIDLSDAI
hDUT            VAPRSGLAAKHFIDVGAGVIDEDYRGNVGVVLFN-------------FGKE---------
                ::.***:::* .: : :* ** .*:**:*: : *             *. :

                       130       140       150       160       170
                         |         |         |         |         |
Φ11DUT          RQYGTYQINEGDKLAQLVIVPIWTPELKQVEEFESVSERGEKGFGSSGV-
hDUT            ----KFEVKKGDRIAQLICERIFYPEIEEVQALDDT-ERGSGGFGSTGKN
                    .:::::**::***:   *: **:::*: ::.. ***. ****:*


                        10        20        30        40        50        60
                         |         |         |         |         |         |
80α             MTNTLQVKLLSKNARMPERNHKTDAGYDIFSAETVVLEPQEKAVIKTDVAVSIPEGYVGL
hDUT            MQ--LRFARLSEHATAPTRGSARAAGYDLYSAYDYTIPPMEKAVVKTDIQIALPSGCYGR
                *   *:.  **::*  * *.    ****::**   .: * ****:***: :::*.*  *

                        70        80        90       100       110       120
                         |         |         |         |         |         |
80α             LTSRSGVSSKTHLVIETGKIDAGYHGNLGINIKNDHEDDKMQTIFLRNIDNEKIFEKERH
hDUT            VAPRSGLAAKHFIDVGAGVIDEDYRGNVGVVLFN--------------------FGKEK-
                ::.***:::* .: : :* ** .*:**:*: : *                    * **:

                       130       140       150       160       170
                         |         |         |         |         |
80α             LYKLGSYRIEKGERIAQLVIVPIWTPELKQVEEFESVSERGEKGFGSSGV-
hDUT            ------FEVKKGDRIAQLICERIFYPEIEEVQALDDT-ERGSGGFGSTGKN
                      :.::**:*****:   *: **:::*: ::.. ***. ****:*
```

Figure S7. Alignment of different human dUTPase with phage Φ11 and 80α phage dUTPases. Identical residues are denoted with stars (*). Strongly similar residues are denoted with colons (:), weakly similar residues are denoted by dots (.).

Supplemental Tables

Table S1. Clusters of the top 20 models for $hDUT_3Stl_3$ obtained from SAXS and HDX-MS data and χ^2 statistics of the obtained models. Clustering was made based on the relative position of the N-terminal (residues 1-84, StlN) and C-terminal (residues 85-267, StlC) segments of Stl to the dUTPase substrate binding pocket. dUTPase (trimer) surface is coloured by grey, substrate analogue is shown as sticks with atomic cloring (carbon black, nitrogen blue, oxigen red, phosphorus orange), Stl models are shown as ribbon with different colors. Note that for ease visibility only one out of the 3 Stls from $hDUT_3Stl_3$ is shown. Since substrate and Stl can not simultaneously bind to the dUTPase, substrate analogue is shown only to designate the substrate binding pocket. Based on HDX-MS and other experimental evidence StlC directly interacts the dUTPase, while StlN has a limited contribution to Stl-dUTPase interaction [3].

**Cluster**	**Models**	χ^2	**Relative orientation of Stl domains to hDUT**
1. 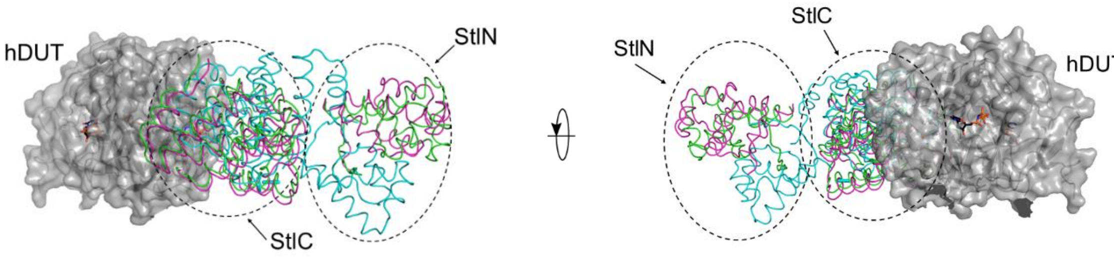	11 (green)<br>12 (cyan)<br>17 (magenta)	**1.4**<br>2.3<br>**1.4**	C-term close,<br>N-term points out<br>1 active site is blocked
2. 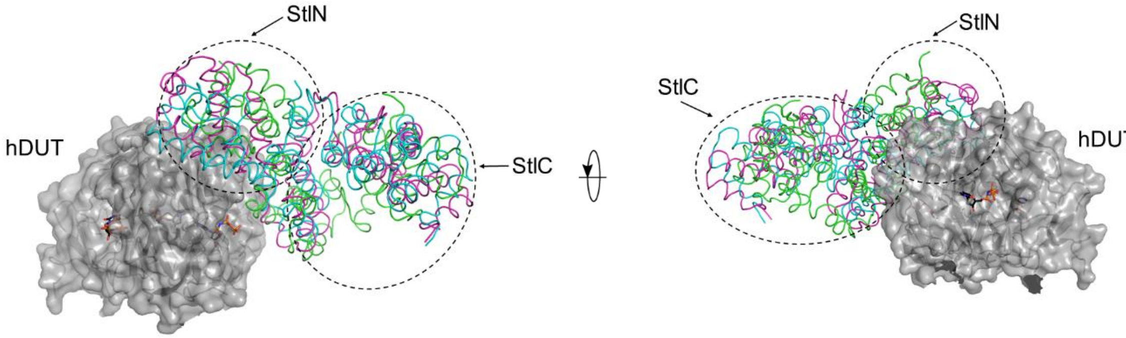	1 (green)<br>16 (cyan)<br>20 (magenta)	1.8<br>2.0<br>1.6	most of C-term points out,<br>N-term contacts DUT<br>zero active site is blocked

Cluster	Models	χ^2	Relative orientation of Stl domains to hDUT
3. 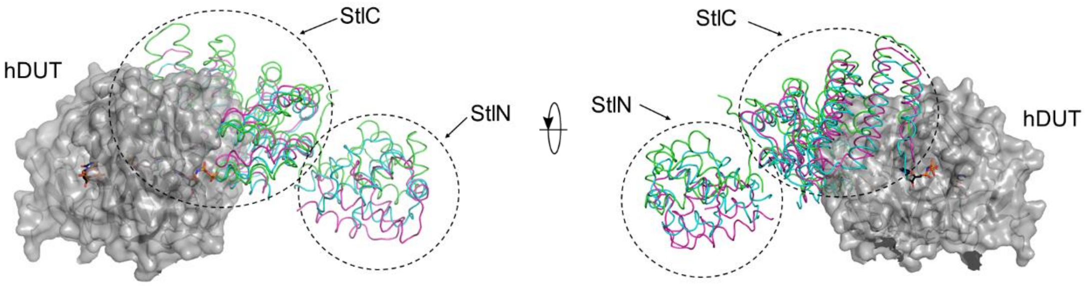	2 (green)	1.7	C-term close,
	10 (cyan)	1.5	N-term points out
	14 (magenta)	1.5	zero active site is blocked
4. 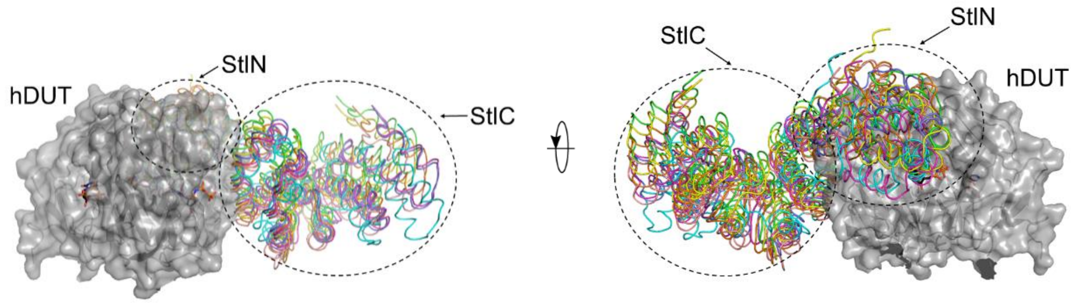	3 (green)	1.8	most of C-term points out,
	5 (cyan)	1.7	N-term contacts DUT
	6 (magenta)	1.8	zero active site is blocked
	8 (yellow)	1.6	
	9 (salmon)	1.7	
	13 (purple)	2.0	
	18 (orange)	1.8	
5. 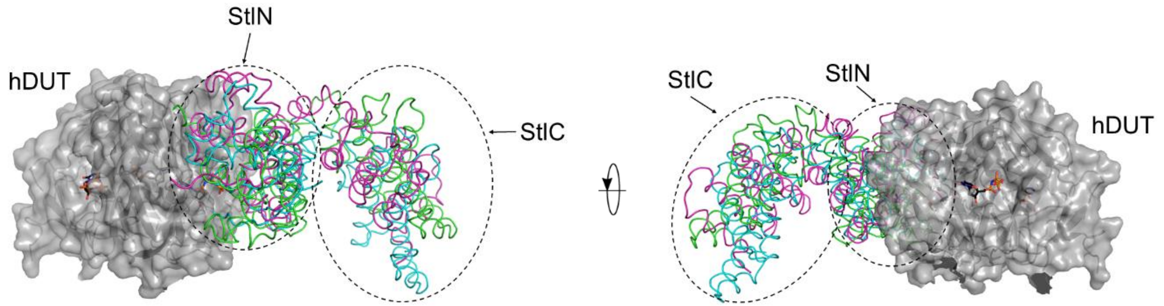	7 (green)	1.8	most of C-term points out,
	15 (cyan)	2.1	N-term contacts DUT
	19 (magenta)	1.8	1 active site is blocked

Cluster	Models	χ^2	Relative orientation of Stl domains to hDUT
6. 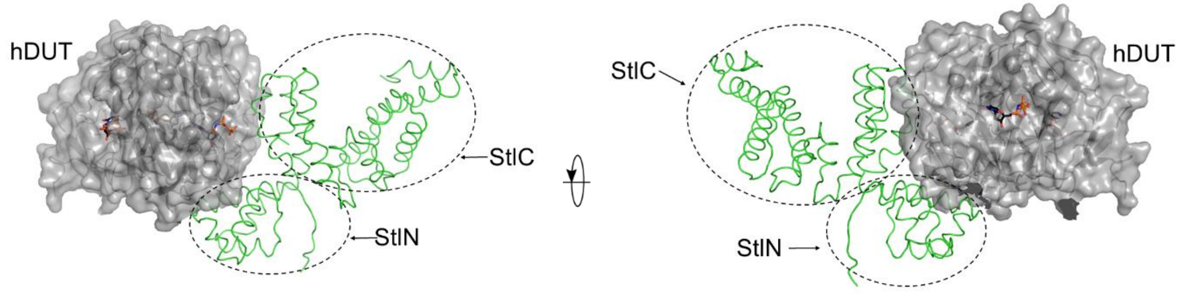	4 (green)	2.2	most of C-term points out,<br>N-term contacts DUT<br>zero active site is blocked

Supplemental References

1. Szabó, J. E. *et al.* Highly potent dUTPase inhibition by a bacterial repressor protein reveals a novel mechanism for gene expression control. *Nucleic Acids Res.* **42,** 11912–20 (2014).

2. Róna, G. *et al.* NLS copy-number variation governs efficiency of nuclear import--case study on dUTPases. *FEBS J.* **281,** 5463–78 (2014).

3. Nyíri, K. *et al.* Evidence-Based Structural Model of the Staphylococcal Repressor Protein: Separation of Functions into Different Domains. *PLoS One* **10,** e0139086 (2015).

Full-length gel of **Figure 1A** and **B**

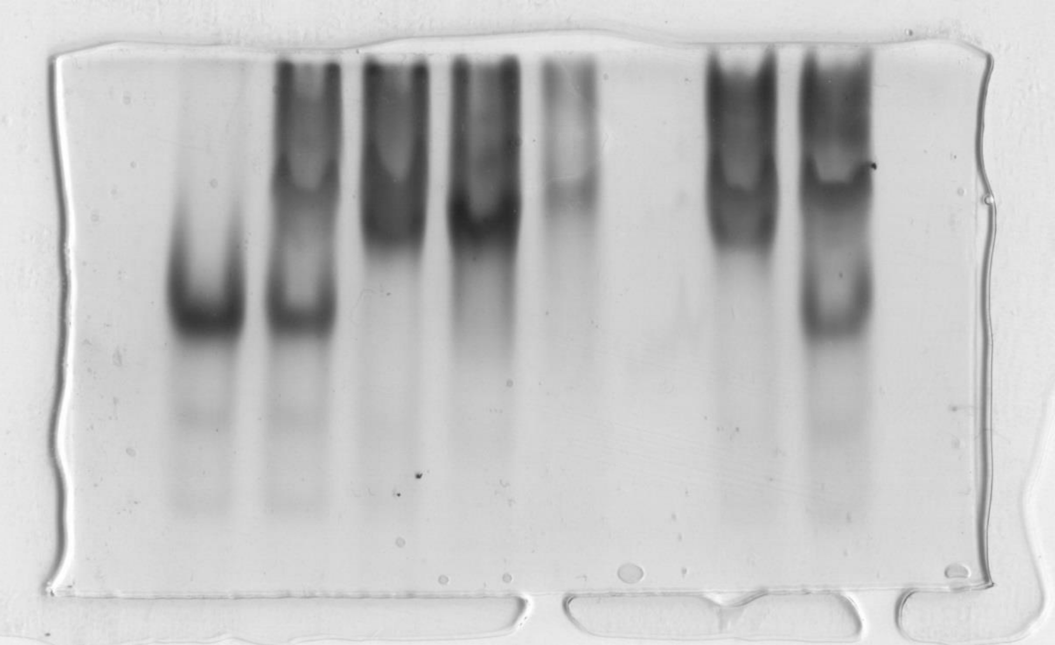

Full-length gel of **Figure 1D**

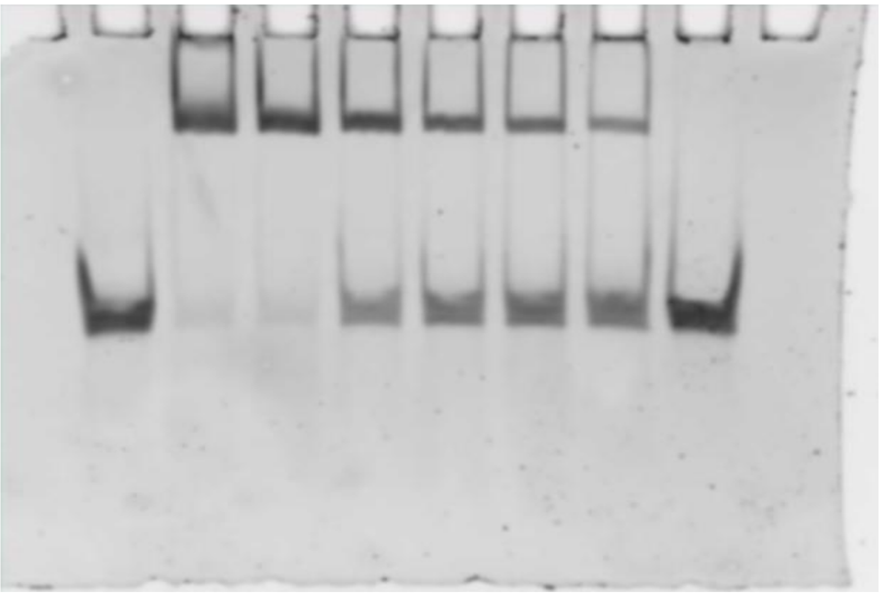

Supplement: Supplementary file 1 — Supporting Information [file 41598_2018_22145_MOESM1_ESM.pdf]
